# Spatially Resolved Multiomics Reveals Metabolic Remodeling and Autophagy Activation in Adamantinomatous Craniopharyngiomas

**DOI:** 10.1002/advs.202516965

**Published:** 2026-01-05

**Authors:** Dongting Chen, Yahui Gao, Yulin Wang, Ting Lei, Zheng Qu, Yuhan An, Jiaxu Fu, Xin Li, Fangjun Liu, Yan Li

**Affiliations:** ^1^ Beijing Key Laboratory of New Drug Mechanisms and Pharmacological Evaluation Study Department of Pharmacology Institute of Materia Medica Chinese Academy of Medical Sciences and Peking Union Medical College Beijing China; ^2^ State Key Laboratory of Bioactive Substance and Function of Natural Medicines Institute of Materia Medica Chinese Academy of Medical Sciences and Peking Union Medical College Beijing China; ^3^ Department of Neurosurgery Sanbo Brain Hospital Capital Medical University Beijing China

**Keywords:** adamantinomatous craniopharyngioma, glycerophospholipid metabolism, multiomics, single‐cell spatial transcriptomics, spatially resolved metabolomics

## Abstract

Adamantinomatous craniopharyngioma (ACP), a benign yet highly recurrent and therapy—resistant intracranial tumor, remains a considerable clinical challenge because of its complex pathological structure, infiltrative growth, and limited treatment options. Here, integrated spatially resolved multiomics is employed—including single‐cell spatial transcriptomics via CosMx SMI and spatially resolved metabolomics via AFADESI‐MSI, accompanied by bulk metabolomics and functional validation—to unravel the driving factors of ACP progression and recurrence. Analysis results reveal three interdependent biological hallmarks: first, the spatial segregation and molecular heterogeneity of 10 distinct tumor epithelial cell subpopulations within the ACP, each of which presents unique transcriptional signatures; second, in tumor regions and recurrent tumor epithelium tissues, stronger transporter‐mediated choline/ethanolamine uptake from cystic fluid and significant upregulation of phosphatidylcholine (PC) and phosphatidylethanolamine (PE) synthesis is observed, creating the enhanced “cystic fluid–tumor cell” and “choline/ethanolamine–PC/PE” metabolic axis, and demonstrating the spatial metabolic remodeling of ACP; and third, this metabolic axis directly couples to autophagy activation of corresponding regions in ACP tissue, which is validated by multi‐immunohistochemistry for Beclin1 and GABARAP. Together, these findings reveal metabolic remodeling and autophagic activation as critical drivers of ACP progression and recurrence and provide an opportunity for precise biomarker‐driven treatment of this intractable tumor.

## Graphical abstract

Multiomics integration analysis reveals the “cystic fluid–tumor cell” metabolic coupling that mediates active choline/ethanolamine uptake of tumor cells from cystic fluid and PC/PE synthesis pathways reprogramming that mediating autophagy pathway activation within ACP.

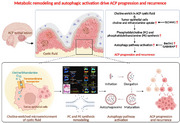

## 1 Introduction

Adamantinomatous craniopharyngiomas (ACPs) are rare intracranial tumors localized in the intrasellar or suprasellar region and are diagnosed with a bimodal peak in incidence (5–15 and 45–60 years) [1]. Moreover, the ACP is the most common benign tumor in the sellar region of children [2]. ACP originates from embryonic craniopharyngeal duct epithelium remnants, which are driven by somatic mutations in CTNNB1 (encoding β‐catenin) exon 3, leading to excessive activation of the WNT pathway [1, 3]. Although ACP has a low degree of histological malignancy (WHO grade I) [4], tumor development is often accompanied by severe complications. Headache, visual impairment, growth retardation, endocrine deficiencies, and hypothalamic syndrome are major symptoms. The prognosis is frequently impaired owing to the hypothalamic–pituitary location and the invasion of surrounding structures. Therefore, it poses considerable challenges for clinical treatment.

The high recurrence rate of ACP is currently a major problem in clinical practice. The first‐line treatments for ACP are neurosurgery and radiotherapy [5]. The primary clinical cause of ACP recurrence is the presence of residual tumor tissues following surgical resection [2, 6, 7]. To control ACP recurrence, in addition to advancements in surgical techniques, the present research also focused on discovering molecular targets and precise treatments. Various dysregulated pathways play roles in the pathogenesis of ACP, which has prompted the investigation of novel treatments [8]. CTNNB1 mutations are the most important marker of ACP. However, inhibition of the Wnt pathway in ACP has not achieved the desired therapeutic effect, and potential off‐target effects may exist [8, 9]. Currently, treatment explorations have focused mainly on the inflammatory and immune microenvironments [10, 11, 12] and the aging phenotype of ACP [13], including the inhibition of interferon‐2α (IFN‐2α), interleukin 6 (IL‐6), and programmed cell death protein 1 and its ligand (PD‐1/PD‐L1) [14, 15, 16]. In addition, overactivation of the MAPK/ERK pathway, SHH pathway, and downstream genes has shown promise as a clinical strategy [17, 18, 19]. However, the efficacy of most of the above treatments is limited, with relatively few cases, inconsistent outcomes, and limited safety [2]. Therefore, further investigations into the underlying mechanism of the development and recurrence of ACP are urgently needed to identify additional potential therapeutic targets.

However, owing to the heterogeneity of ACP tumors, basic research is challenging. The cellular diversity of ACP has been elucidated in previous studies through transcriptomics approaches [20, 21, 22]. Most ACPs have solid–cystic structures [1]. Cysts contain oily fluid rich in cholesterol, lipids, and proinflammatory factors [10, 23]. The solid tumor parts have complex epithelial structures that form palisade‐like epithelium (PE), squamous‐like epithelium (SE), stellate reticulum (SR) cells, whorl‐like epithelium (WE), and wet keratin‐like epithelium (KE). Furthermore, ACP also exhibits distinctive characteristics in terms of tumor metabolism. A previous study revealed that cystic fluid production may be related to lipid metabolism disorders in ACP tumor cells [24]. Another study revealed that increased purine metabolism and redox imbalance may mediate the development of ACP‐associated hypothalamic comorbidities [25].

Currently, the advancement of multiomics technologies, especially spatial omics, has increased the number of opportunities for discovering the molecular features and therapeutic targets of ACP. Two main platforms, CosMx spatial molecular imaging (SMI) via NanoString and Xenium via 10x Genomics, enable spatial transcriptomic assessment at single/sub‐cell resolution [26, 27]. CosMx SMI can accurately visualize and quantitatively analyze the spatial in situ information of numerous targets, which enables researchers to analyze cell heterogeneity and cell‒cell communication in depth and discover important biomarkers [26, 28]. Moreover, the development of spatial metabolomics and spatial lipidomics, including matrix‐assisted laser desorption ionization (MALDI)‐based mass spectrometry imaging (MSI) and desorption electrospray ionization (DESI)‐based MSI technologies, has facilitated in‐depth exploration of the metabolic microenvironment of tumors and their remodeling [29, 30, 31]. However, the roles of the metabolic microenvironment and transcriptional heterogeneity in ACP tumor progression and recurrence remain poorly understood, and an obvious gap exists in the integration of single‐cell spatial transcriptomics and spatially resolved metabolomics to reveal the links between gene expression and metabolic remodeling in different regions. Moreover, the metabolic crosstalk between ACP solid tumors and cystic fluid, which serves as a dynamic metabolic niche, remains poorly characterized. Therefore, we expect to decipher ACP pathogenesis and recurrence mechanisms by integrating multiomics and to elucidate the spatial interplay and networks between cells and tumor‐cyst structures, thereby providing insights into the identification of novel therapeutic targets.

In this study, we used multiomics approach to analyze paired clinical samples, including primary and recurrent ACP tissues, cystic fluids and matched plasma, and sought to 1) characterize the spatial distribution and transcriptional landscapes of heterogeneous cell populations within ACP; 2) decode metabolic remodeling in the tumor and tumor microenvironment (TME), especially cyst; and 3) identify key molecular pathways driving ACP progression and recurrence by linking single‐cell spatial transcriptomic signatures with metabolic alterations. This work not only fills the methodological gap in spatial omics integration in ACP, but also provides mechanistic insights into how tumor heterogeneity and metabolic microenvironments fuel ACP progression and recurrence, as well as provides targets and strategies for ACP treatment.

## 2 Results

### 2.1 Single‐Cell Spatial Profiling of ACP

To reveal the tumor heterogeneity and cellular diversity in ACP, as well as the transcriptomic characteristics of primary and recurrent samples, 10 FFPE samples, including 4 paired primary and recurrent ACPs (ACP_1P/ACP_1R‐ ACP_4P/ACP_4R), 1 primary and 1 recurrent nonpaired ACPs (ACP_6P, ACP_5R), were included in the study. The detailed sample information is shown in Table S1.

**FIGURE 1:**
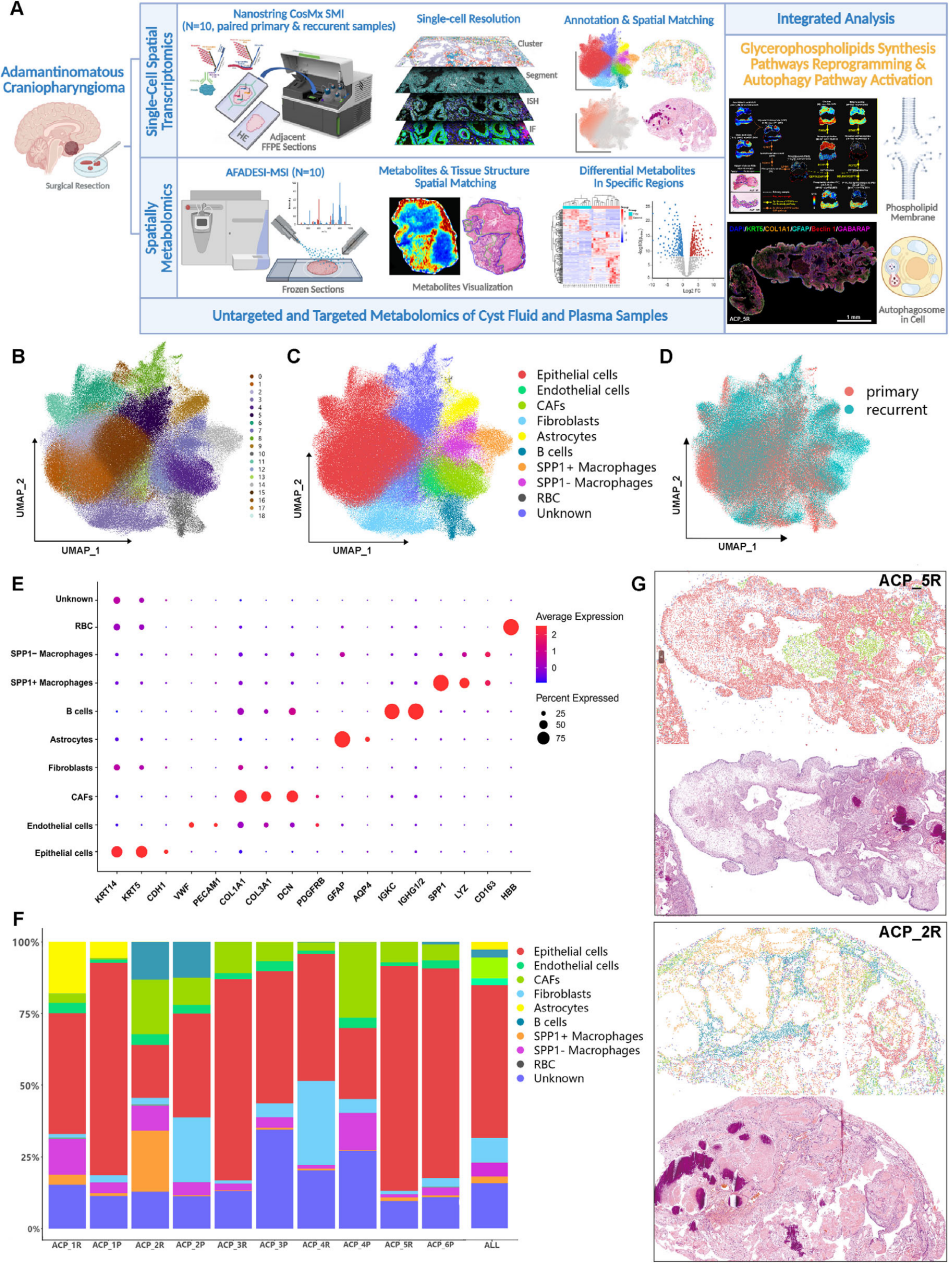
Single‐cell spatial transcriptome sequencing of ACP via the CosMx spatial molecular imaging 6000‐plex human mRNA panel. (A) Schematic overview of the workflow for single‐cell spatial transcriptomics integrated with spatially resolved metabolomics and bulk metabolomic analyses. (B, C) UMAP plots of 296,304 cells visualize the clustering and annotation results on the basis of the single‐cell spatial transcriptomics data. (D) UMAP plots representing the spatial distributions of the cells from the primary and recurrent samples. (E) Dot plots showing the expression levels and proportions of specific marker genes among 10 major cell populations. (F) Bar charts depicting the overall and individual case‐specific cellular composition ratios. P: primary samples; R: recurrent samples. ACP_1P/ACP_1R‐ ACP_4P/ACP_4R are four paired (from the same patient) samples, ACP_6P and ACP_5R are nonpaired samples. (G) Single‐cell population spatial distribution images (partial) corresponding to the pathological structures of adjacent H&E‐stained sections. Representative samples: ACP_5R and ACP_2R. Each dot represents a cell, and the legend (colors representing cell types) is the same as that in (C) and (F).

Single‐cell spatial transcriptome sequencing was conducted with the CosMx SMI in situ molecular imaging platform (Figure 1A). First, the quality of the CosMx SMI instrument run was determined, with low background levels, low rates of nonspecific binding, low negative probes, and false codes (Table S4). The IF images of the morphological markers (DAPI, CD298, pan‐CK, and CD45) used for single‐cell recognition and segmentation, as well as the FOV positions, are shown in Figure S1. After filtering out low‐quality cell populations, 296,304 cells were eventually obtained among 386 FOVs, with 609.9 transcripts detected per cell (Table S4). Next, dimensionality reduction and unsupervised clustering analysis were performed via UMAP, and 19 cell clusters were initially identified (Figure 1B). Finally, 10 main cell populations were annotated on the basis of specific gene markers: epithelial cells marked by KRT14, KRT5, and CDH1; endothelial cells marked by VWF, PECAM1, and PDGFRB; tumor‐associated fibroblasts (CAFs) marked by COL1A1, COL3A1, DCN, and PDGFRB; fibroblasts marked by COL1A1 and COL3A1; astrocytes marked by GFAP and AQP4; B cells marked by IGKC and IGHG1/2; SPP1+ macrophages; and SPP1− macrophages characterized by LYZ and CD163 expression, with high and low SPP1 expression, respectively; and red blood cells (RBC) marked by HBB (Figure 1C,E). UMAP plots were generated to show the spatial distributions of the cells from the primary and recurrent samples (Figure 1D). Feature plots depicting the activity and distribution of marker genes for each cell population are shown in Figure S2. The most abundant cell type in the overall samples (average value) was epithelial cells, accounting for approximately 50%, followed by fibroblasts, CAFs, and macrophages (Figure 1F).

In the cell annotation results on the spatial in situ map, the spatial distribution of single‐cell populations was in excellent agreement with the pathological structures shown in the hematoxylin and eosin (H&E)‐stained adjacent slides (Figures 1G and S3). In ZOOM1 of ACP_1P, the junction between the tumor epithelial tissue and the glial scar was observed. Interestingly, keratin nodules were distributed within the glial scar and were surrounded by abundant macrophages (Figure S3). This characteristic might be caused by the inflammation resulting from cell necrosis during keratin nodule formation (the nucleus of the keratin‐like epithelium continuously shrank along with cytolysis, eventually forming a dead cell mass), which in turn attracted immune cells. This colocalization also occurred in the ZOOM2 domain of ACP_2R. Large areas of keratin nodules were surrounded by numerous macrophages (with more SPP1+ macrophages) and B cells (Figure S3). ZOOM3 of ACP_4R and ZOOM4 of ACP_5R together showed that CAFs and fibroblasts interweaved with the tumor epithelium, whereas endothelial cells formed vascular structures scattered throughout the tumor epithelium (Figure S3).

To further dissect the functional diversity of single‐cell populations and their coordinated roles in shaping the ACP TME, spatial co‐expressed gene modules were identified, which are sets of genes that tend to be expressed in the same tissue regions (Figure S4). Heatmap showed score values for each co‐expressed gene module in cell populations, tumor epithelial cells exhibited distinct spatial gene co‐expression patterns from other cell populations (Figure S4A). The single cell scores of the top 5 modules (APOE_PSAP_52, DCN_COL1A2_30, FOS_FOSB_29, KRT5_KRT19_29, and A1BG_IL22RA2_14, ranked by number of genes included) were mapped onto spatial in situ heatmaps (Figure S4B–F). Gene sets included in the top 5 modules conducted functional pathway enrichment by Gene Ontology (GO), respectively (Figure S5). APOE_PSAP_52 had higher module scores in macrophages and CAFs, enriched in immune system processes, consistent with the dominant role of macrophages in the ACP TME. DCN_COL1A2_30 showed higher module scores in CAFs, fibroblasts, and endothelial cells, extracellular matrix organization and tissue development were enriched, corresponding to stromal cell functions. FOS_FOSB_29 and KRT5_KRT19_29 gained highest module scores in epithelial cells, with active pathways of RNA metabolic processes, tissue development, epithelial cell and keratinocyte differentiation, aligning with the active proliferation and keratinization characteristics of tumor epithelium. A1BG_IL22RA2_14 had highest module scores in fibroblasts, enriched in peptidyl‐tyrosine phosphorylation, aging, and ATP synthesis coupled electron transport.

Overall, ACP tumor heterogeneity and transcriptional characteristics can be precisely interpreted at the single‐cell spatial level.

### 2.2 Single‐Cell Spatial Transcriptomics Revealed the Complexity and Positioning Features of ACP Tumor Epithelial Cell Subpopulations

**FIGURE 2:**
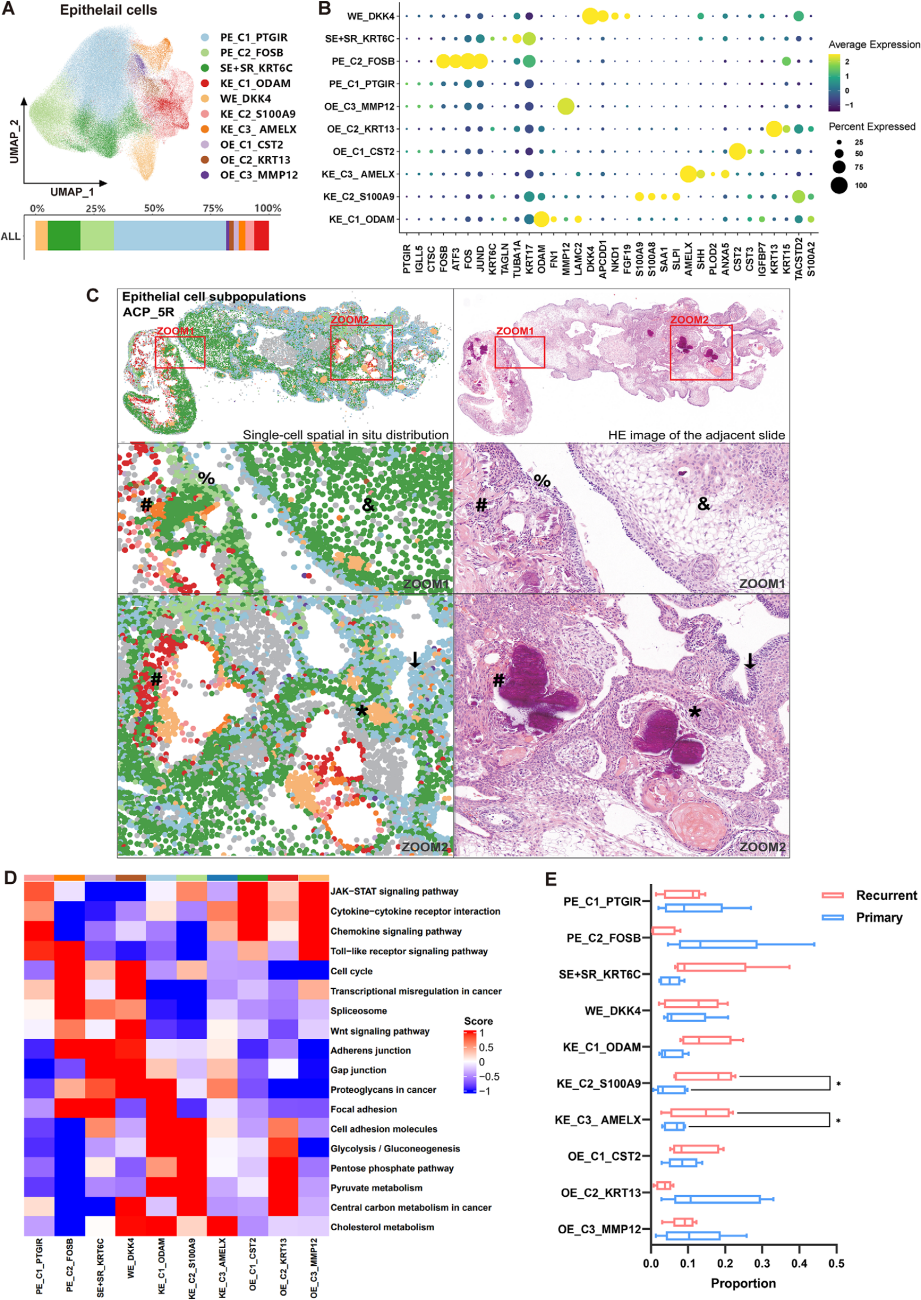
Reclustering revealed distinct subpopulations of tumor epithelial cells in ACP. (A) UMAP plots showing the 10 cellular subpopulations of the tumor epithelial cells. Bar charts depicting the overall cellular composition ratios in 10 samples. (B) Dot plots showing the gene expression features of 10 tumor epithelial cell subpopulations. (C) Spatial distribution images of tumor epithelial cell subpopulations corresponding to the pathological structures in adjacent H&E‐stained sections. Representative sample: ACP_5R; each dot represents a cell, the legend is the same as that in (A), gray dots represent non‐epithelial cells. ↓ and %: palisade‐like epithelium (PE), &: squamous‐like epithelium (SE) and stellate reticulum cells (SR), *: whorl‐like epithelium (WE), #: wet keratin‐like epithelium (KE). (D) Heatmaps showing the activity scores of different pathways in the KEGG database between cellular subpopulations scored by GSVA. (E) Box‐whisker plots reveal the proportions of tumor epithelial cell subpopulations in recurrent samples (*n* = 10) vs. primary samples (*n* = 10). *p* values were calculated via the Student's *t*‐test, *p < 0.05.

In order to resolve tumor epithelial cell subpopulations more accurately at single‐cell resolution, and to locate the distribution characteristics of different subpopulations more precisely, the tumor epithelial cell population was extracted and subjected to reclustering. The grouping of tumor epithelial cells identified 10 clusters: PE_C1_PTGIR, PE_C2_FOSB, SE+SR_KRT6C, WE_DKK4, KE_C1_ODAM, KE_C2_S100A9, KE_C3_AMELX, OE_C1_CST2, OE_C2_KRT13, and OE_C3_MMP12, with PE_C1_PTGIR accounting for the largest proportion (Figure 2A,B). The in situ distribution maps of tumor epithelial cell subpopulations correspond well with the heterogeneous pathological structures of the epithelium shown by H&E‐stained adjacent sections (Figure 2C).

Take ACP_5R as an example. PE_C1_PTGIR (light blue spots, areas marked by arrows) and PE_C2_FOSB (light green spots, areas marked by percentages) clusters constituted the palisade‐like epithelium (PE) structures. Although the two subpopulations of cells presented no obvious morphological differences, they presented distinct gene expression patterns (Figure 2B,C). The PE_C1_PTGIR cluster presented increased expression levels of PTGIR, IGLL5, and CTSC, which are related to immune system regulation or the inflammatory response (Figure 2B). Similarly, the gene set variation analysis (GSVA) scores for the KEGG pathways revealed that the JAK–STAT signaling pathway, the cytokine–cytokine receptor interaction, and the chemokine signaling pathway were significantly enriched in PE_C1_PTGIR (Figure 2D). The genes in the PE_C2_FOSB cluster were strongly related to the stress response (oxidative stress, DNA damage, inflammatory factors, etc.), cell proliferation and the regulation of apoptosis, such as FOS, FOSB, JUND, and ATF3 (Figure 2B). GSVA revealed the enrichment of the Toll‐like receptor signaling pathway, the cell cycle, the transcriptional misregulation in cancer, and the spliceosome, which reflected the inflammatory response and abnormal proliferation ability of the PE_C2_FOSB cluster (Figure 2D).

The SE+SR_KRT6C (dark green spots, area marked by the ampersand) and WE_DKK4 (yellow spots, area marked by an asterisk) clusters corresponded to the squamous‐like epithelium (SE), stellate reticulum (SR) cells, and whorl‐like epithelium (WE), respectively (Figure 2C). The SE+SR_KRT6C cluster presented high expression of KRT6C, KRT17, TAGLN, and TUBA1A, which may reflect the characteristics of stratified squamous epithelium or basal epithelium and is closely linked to the high dynamics, migration, and repair functions of cells. The WE_DKK4 cluster strongly expressed genes regulating the Wnt signaling pathway, such as DKK4, APCDD1, and NKD1. Interestingly, all three genes were negative regulators of the Wnt signaling pathway (Figure 2B). The GSVA scores were also consistent with the gene expression features described above. Pathways related to cell adhesion, intercellular junctions, and proteoglycans in cancer, which are associated with cell migration and tissue repair, were significantly upregulated in the SE+SR_KRT6C cluster. The Wnt signaling pathway had the highest score in the WE_DKK4 cluster (Figure 2D).

Wet keratin‐like epithelium (KE) is also considered a typical structure of the ACP. The three clusters of cells, KE_C1_ODAM, KE_C2_S100A9, and KE_C3_AMELX (red, pink and orange spots, respectively, areas marked by well numbers), were located in the KE area. The spatial distributions of these three clusters overlapped considerably, with no obvious morphological differences, and functional interactions may occur. These three KE clusters often colocalized with WE, keratin nodules, and dystrophic calcification (calcium salt deposition caused by local tissue lesions) (Figure 2C). However, they also exhibited significant transcriptional differences (Figure 2B). In addition, the other three types of epithelial cell clusters, OE_C1_CST2, OE_C2_KRT13, and OE_C3_MMP12, were scattered randomly throughout the tissue, without corresponding epithelial structural features in the H&E‐stained sections. Notably, pathways related to energy metabolism, such as glycolysis/gluconeogenesis and pentose phosphate pathways, were significantly enriched in the KE_C1_ODAM, KE_C2_S100A9, and OE_C2_KRT13 clusters, whereas the cholesterol metabolism pathway was enriched in the WE_DKK4 and three KE clusters (Figure 2D). These findings suggest that different epithelial cell subpopulations within ACP exhibited diverse metabolic profiles. In addition, the proportions of tumor epithelial cell subpopulations in the primary and recurrent samples also exhibited heterogeneity. As shown in Figure 2E, the KE_C2_S100A9 and KE_C3_AMELX clusters accounted for a significantly greater proportion of the recurrent samples.

### 2.3 Spatially Resolved Metabolomics Landscape of ACP

To understand metabolic remodeling within the ACP TME, spatially resolved metabolomics analysis was conducted on 10 ACP samples, including 5 primary samples and 5 recurrent samples (nonpaired) (ACP_6P‐ACP_10P; ACP_5R, ACP_11R‐ ACP_14R). The detailed sample information is shown in Table S1. Moreover, integrated analysis was performed with single‐cell spatial transcriptomics data to obtain multilayer molecular information in heterogeneous tumor tissues.

**FIGURE 3:**
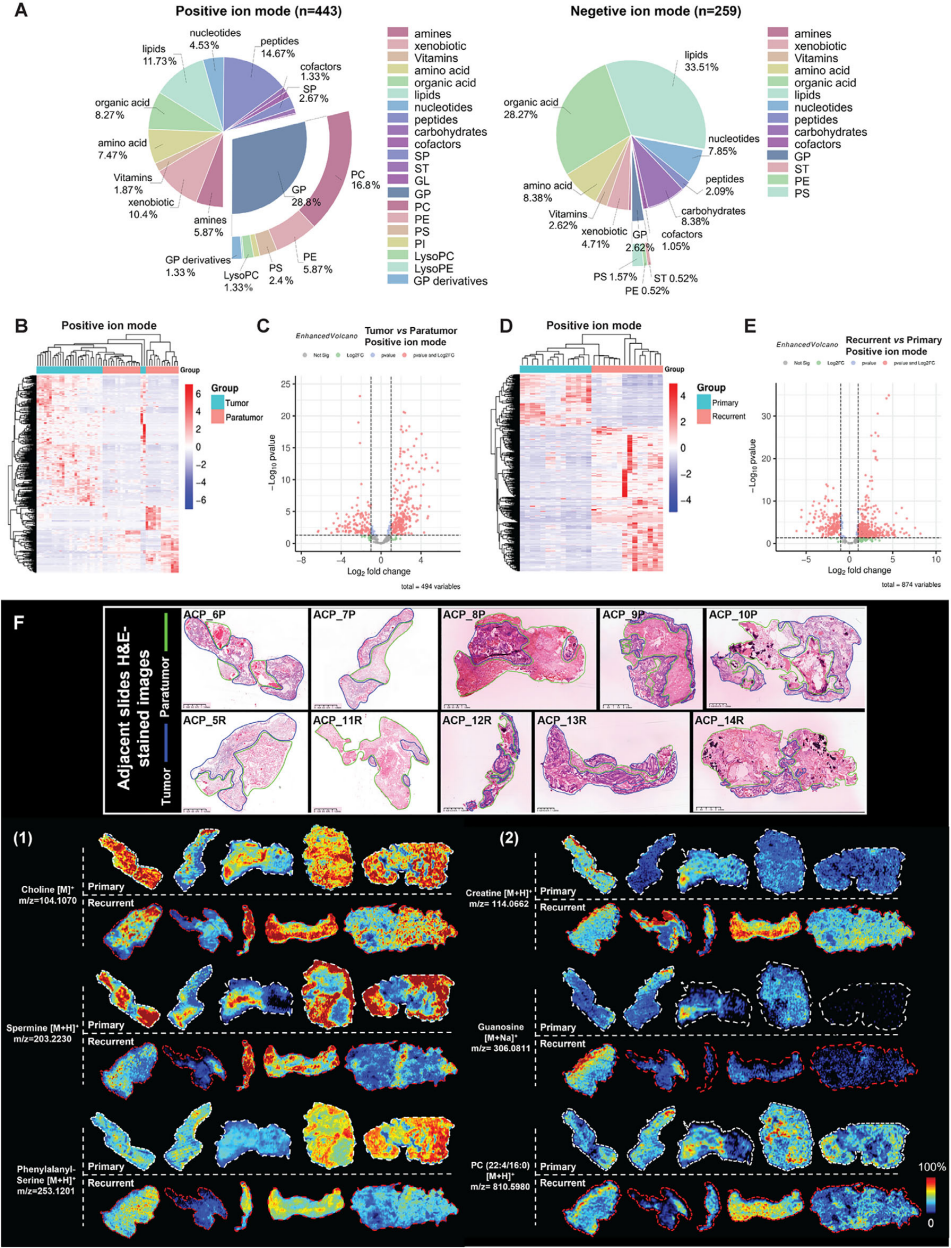
Spatially resolved metabolomics analysis of ACP by AFADESI‐MSI. (A) Classification of 443 and 259 annotated metabolites in positive‐ and negative‐ion mode, respectively. SP, sphingolipid; ST, sterol lipid; GL, glyceride; GP, glycerophospholipid; PC, phosphatidylcholine; PE, phosphatidylethanolamine; PS, phosphatidylserine; PI, phosphatidylinositol. (B) Cluster heatmap of metabolite abundance between the tumor and paratumor tissues in positive‐ion mode (ROIs were selected from the 10 samples, *n* = 14 each group). (C) Volcano plots showing 494 differentially enriched metabolites (VIP > 1 in OPLS‐DA) in positive‐ion mode between the tumor and paratumor tissues. A total of 252 and 118 metabolites were significantly upregulated and downregulated (*p* < 0.05 in the Mann–Whitney *U* test, |log2FC| > 1) in tumor tissues, respectively. (D) Cluster heatmap of metabolite abundance between the primary and recurrent tumor epithelium tissues in positive ion mode (ROIs were selected from the 10 samples, *n* = 14 each group). (E) Volcano plots showing 874 differentially enriched metabolites (VIP > 1 in OPLS‐DA) in positive‐ion mode between the primary and recurrent tumor epithelium tissues. A total of 542 and 184 metabolites were significantly upregulated and downregulated (*p* < 0.05 in the Mann–Whitney *U* test, |Log2FC| > 1) in recurrent tumor epithelium tissues, respectively. (F) Ion images of representative differentially abundant metabolites in ACP (intensity in color scale is relative value).

Spatially resolved metabolomics was conducted via AFADESI‐MSI (Figure 1A). The raw data were preprocessed, and metabolite annotation was performed. Finally, a total of 443 and 259 metabolites was annotated in positive‐ and negative‐ion mode, respectively (Figure 3A). In the positive ion mode, glycerophospholipids (GPs) were the most abundant classes of metabolite, followed by peptides and lipids. GPs included phosphatidylcholine (PC), phosphatidylethanolamine (PE), phosphatidylserine (PS), phosphatidylinositol (PI), lysoPC, lysoPE, and GP derivatives. Whereas in the negative‐ion mode, lipids and organic acid were the top two enriched classes of metabolite.

To reveal the metabolic characteristics of ACP, different types of regions of interest (ROIs) were selected from ACP tissues and grouped for analysis. We selected 14 ROIs for each of the tumor tissue, paratumor tissue, primary tumor epithelium tissue and recurrent tumor epithelium tissue from 10 samples. A cluster heatmap of metabolite abundance and principal component analysis (PCA) revealed a clear clustering and grouping trend between the tumor tissues and paratumor tissues, and most metabolites tended to increase in tumor tissues (Figures 3B and S6A,B). Next, orthogonal partial least‐squares discriminant analysis (OPLS‐DA) and the Mann–Whitney *U* test were used to screen differentially abundant metabolites (variable importance in the projection (VIP) > 1, *p* < 0.05, and |Log2FC| > 1), and 542 metabolites (summation of positive‐ and negative‐ion modes; 390 upregulated and 152 downregulated in tumor tissues) with significant differences in abundance between tumor and paratumor tissues were identified (Figures 3C and S6C,G). Moreover, differentially abundant metabolites between primary and recurrent tumor epithelial tissues were identified via the same screening mode. There was also a good clustering and grouping trend between primary and recurrent tumor epithelium tissues, and most metabolites tended to increase in recurrent tumor epithelium tissues (Figures 3D and S6D,E). Finally, 910 metabolites (summation of positive‐ and negative‐ion modes; 674 upregulated and 236 downregulated in recurrent tumor epithelium tissues) displayed significant differences in abundance between primary and recurrent tumor epithelium tissues (Figures 3E and S6F,H).

Ion images of representative differentially abundant metabolites are shown in Figure 3F. Most metabolites exhibit highly heterogeneous spatial characteristics. Organic acids, organic bases, polyamines, nucleotides, amino acids, peptides, phospholipids, and fatty acids were annotated to have significant difference between tumor tissues and paratumor tissues. The ion images of choline (organic base), spermine (polyamine), and phenylalanine‐serine (peptide) are shown as representative examples (Figure 3F (1)). Similarly, metabolites of the above types also significantly differed between primary and recurrent tumor epithelium tissues. The ion images of creatine (organic acid), guanosine (nucleoside), and PC (phospholipid) are shown as representative examples (Figure 3F (2)). Collectively, these differentially abundant metabolites may play indispensable roles in the progression and recurrence of ACP.

### 2.4 PC and PE Synthesis Pathways Remodeling in ACP

**FIGURE 4:**
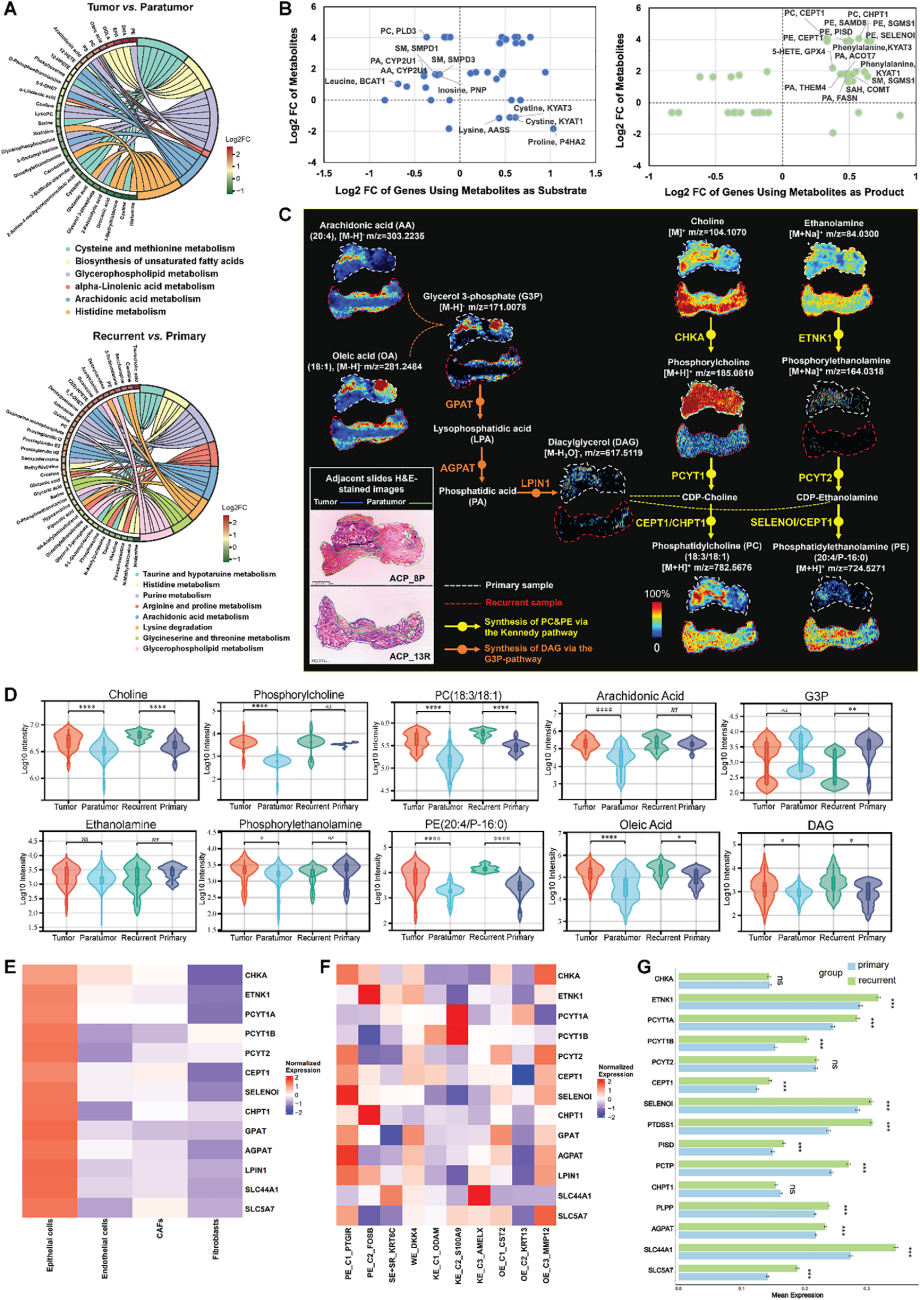
Visualization of phosphatidylcholine (PC) and phosphatidylethanolamine (PE) synthesis pathways reprogramming in ACP. (A) Metabolic pathway enrichment analysis of the differentially abundant metabolites via the MetaboAnalyst and KEGG databases. Above: significantly upregulated pathways in tumor tissues compared with those in paratumor tissues; below: significantly upregulated pathways in recurrent tumor epithelium tissues compared with those in primary tumor epithelium tissues. (B) Correlation of the mRNA expression of metabolic genes with the abundances of paired metabolites as substrates (left) or products (right). Metabolite–gene pairs were derived from the MetaboAnalyst and KEGG databases. Pairs with significant differences between tumor tissues and paratumor tissues are annotated in the plot. (C) Ion images of key metabolites in the PC and PE synthesis pathways (ACP_8P and ACP_13R tissues are shown as representatives); scale bar of H&E‐stained images = 1.25 mm, intensity in color scale is relative value. (D) Ion intensities of key metabolites between tumor and paratumor tissues and between primary and recurrent tumor epithelium tissues (*n* = 14). *p* values were calculated via the Mann–Whitney *U* test. ****p < 0.0001, **p < 0.01, *p < 0.05, ns: nonsignificant. (E, F) Expression heatmap of the PC/PE synthesis pathway and choline/ethanolamine transporter genes between tumor epithelial cells with paratumor cells, and between different epithelial cell subpopulations. (G) Mean expression bar plots of the glycerophospholipid metabolism pathway genes between primary and recurrent tumor epithelial cells. *p* values were calculated via the Student's *t*‐test. ***p < 0.001, ns: nonsignificant.

To further investigate the metabolic heterogeneity of different regions in ACP tissues, as well as the metabolic differences between primary and recurrent tumors, we performed metabolic pathway enrichment of differentially abundant metabolites within the two groups. As shown in Figure 4A, the glycerophospholipid metabolism pathway was significantly activated in tumor tissues and recurrent tumor epithelium tissues. Systematic evaluations were also performed linking differentially abundant metabolites to genomic features (Figure 4B). PC and PE, which are glycerophospholipids, were significantly upregulated in tumor tissues compared with paratumor tissues. Moreover, according to the transcriptomics data, catabolic genes that use PC as substrate, such as PLD3, were downregulated in tumor epithelial cells, whereas PC/PE anabolic genes, such as CEPT1, CHPT1, and SELENOI, were upregulated in tumor epithelial cells. On the basis of the above results, we speculated that glycerophospholipids, especially PC and PE, play important roles in ACP. Next, we further investigated the PC and PE synthesis pathways in ACP.

PC and PE are the two most abundant glycerophospholipids in mammalian cells. In the human body, the biosynthesis of PC and PE occurs mainly through the de novo synthetic pathway, which is the three‐step Kennedy pathway from choline and ethanolamine via phospho‐ and cytidinediphospho‐ (CDP‐) intermediates [32]. Choline kinase alpha (CHKA), ethanolamine kinase 1 (ETNK1), phosphate cytidylyltransferase (PCYT), choline/ethanolamine phosphotransferase 1 (CEPT1), selenoprotein I (SELENOI), and choline phosphotransferase 1 (CHPT1) are the key enzymes in the Kennedy pathway. Diacylglycerol (DAG) acts as a lipid receptor that binds to phosphorylcholine/phosphorylanolamine from CDP‐choline/CDP‐ethanolamine for PC/PE production [33]. Two pathways contribute to the anabolic generation of DAGs: the glycerol 3‐phosphate (G3P) pathway and the MAG pathway. The G3P pathway is the major pathway of DAG synthesis in most tissues and involves the consecutive acylation of G3P to introduce fatty acid chains, which are catalyzed by acyl‐CoA‐dependent glycerol‐3‐phosphate acyltransferase (GPAT) and acyl‐CoA acylglycerol‐3‐phosphate acyltransferase (AGPAT). The product of these reactions is phosphatidic acid (PA), which is dephosphorylated to DAG by PA phosphatase (LPIN1) [34, 35].

The ion images of key metabolites involved in the G3P pathway and Kennedy pathway are shown in Figure 4C (ACP_8P and ACP_13R tissues are shown as representatives). Notably, the ion intensities of saturated/unsaturated PCs and PEs, such as PC (16:0/16:0), PC (18:3/18:1), and PE (20:4/16:0), were significantly increased in tumor tissues than in paratumor tissues and in recurrent tumor epithelium tissues than in primary tumor epithelium tissues (Figures 4A,C,D and S7). Similarly, the raw materials utilized in PC and PE synthesis, that is, choline, ethanolamine, and DAG, and the raw materials used for DAG synthesis, that is, fatty acids (such as arachidonic acid and oleic acid), were also enriched in tumor tissues compared with paratumor tissues and in recurrent tumor epithelium tissues compared with primary tumor epithelium tissues (Figure 4A,C,D). However, the enrichment of ethanolamine did not show statistical significance (Figure 4D). In addition, since phosphorylcholine/phosphorylethanolamine, CDP‐choline/CDP‐ethanolamine, LPA and PA are intermediates in the Kennedy pathway and the DAG synthesis pathway, their enrichment features are ambiguous or not annotated.

According to the single‐cell spatial transcriptomics data, genes involved in the PC/PE synthesis pathways showed marked upregulation in tumor epithelial cells compared with those in paratumor cells, indicated the PC/PE synthetic activation in tumor epithelium tissues (Figure 4E; endothelial cells, CAFs, and fibroblasts were considered as paratumor cells). The expression heterogeneous of these genes within different tumor epithelial subpopulations was further dissected, each subpopulations exhibited distinct expression profiles (Figure 4F). Among them, PE_C1_PTGIR and OE_C3_MMP12 subpopulations showed holistic upregulation of key synthetases, indicating the core contributions to PC/PE synthesis, while genes in the OE_C2_KRT13 subpopulation were relatively downregulated. These results collectively demonstrated that PC/PE synthetic activation was not uniform across the tumor epithelium tissues, but was dominated by specific subgroups. Meanwhile, most of genes involved in the glycerophospholipid metabolism were also upregulated in recurrent tumor epithelial cells compared with primary tumor epithelial cells (Figure 4G). Interestingly, the expressions of SLC44A1 and SLC5A7, which are transmembrane transporters for choline/ethanolamine uptake, were significantly upregulated in tumor epithelial cells and in recurrent tumor epithelial cells (compared with that in paratumor cells and primary tumor epithelial cells, respectively) (Figure 4E,G). This finding could explain the enrichment of choline and ethanolamine in these regions, as ACP tumor cells meet the need for PC and PE synthesis by increasing choline and ethanolamine uptake.

In summary, through the enrichment of metabolites in the spatial metabolomics, as well as the metabolic genes activation in the spatial single‐cell transcriptomics, we combined characterized the hyperactivity of PC and PE synthesis in ACP, which may be a key factor in promoting ACP development and recurrence.

### 2.5 Untargeted and Targeted Metabolomics Revealed a Choline‐Enriched Microenvironment in ACP Cystic Fluid

The significant enrichment of choline was analyzed by spatially resolved multiomics, revealing a key precursor for PC synthesis and the upregulation of the choline transporter SLC44A1 within specific tumor regions. We hypothesized that the characteristic cystic fluid microenvironment of ACP might serve as a significant source of choline. For verification, we conducted untargeted and targeted metabolomics analyses of ACP cystic fluid and paired plasma samples. The detailed sample information is shown in Table S2.

**FIGURE 5:**
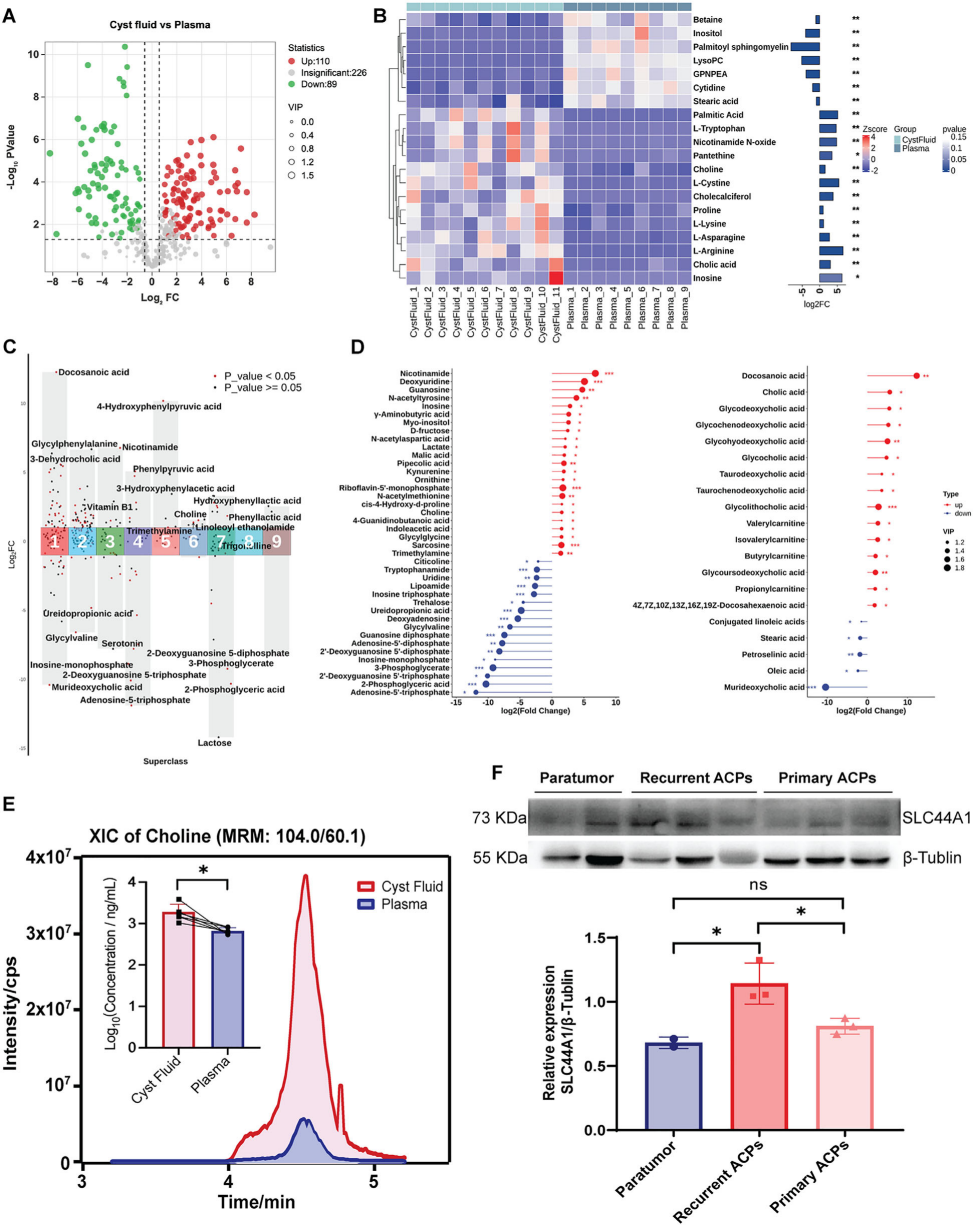
Untargeted and targeted metabolomics were used to analyze metabolites in ACP cystic fluid. (A) Volcano plots showing the 425 metabolites annotated via untargeted metabolomics. Compared with those in paired plasma (*n* = 9), 110 and 89 metabolites in cystic fluid (*n* = 11) were significantly upregulated and downregulated (VIP > 1, *p* < 0.05 in the Mann–Whitney *U* test, |FC| > 1.5), respectively. (B) Cluster heatmap of partial differentially abundant metabolites between cystic fluid and paired plasma samples via untargeted metabolomics. The right side of the figure shows the fold change and significance of the differentially abundant metabolites. (C) A total of 344 metabolites was detected in cystic fluid (*n* = 6) and paired plasma (*n* = 6) via targeted metabolomics, and all the metabolites were divided into 9 categories according to HMDB superclass classification. 1: lipids and lipid‐like molecules; 2: organic acids and derivatives; 3: organo‐heterocyclic compounds; 4: nucleosides, nucleotides, and analogues; 5: benzenoids; 6: organic nitrogen compounds; 7: organic oxygen compounds; 8: alkaloids and derivatives; 9: phenylpropanoids and polyketides. (D) Lollipop charts showing the differentially abundant metabolites of the lipid (left) and nonlipid (right) classes in the targeted metabolomics. (E) Targeted quantification results via LC‐MS/MS show the choline concentration in cystic fluid (*n* = 6) compared with that in paired plasma (*n* = 6). *p* values were calculated via the Mann–Whitney *U* test. *p < 0.05. (F) Western blotting was performed to verify the expression of the SLC44A1 in primary ACPs (*n* = 3), recurrent ACPs (*n* = 3) and paratumor tissues (*n* = 2). The results are shown as mean ± SD; *p* values were calculated via the one‐way ANOVA. *p < 0.05, ns: nonsignificant.

In untargeted metabolomics, 425 metabolites were detected and annotated, among which 110 and 89 metabolites were significantly upregulated and downregulated, respectively, in cystic fluid compared with paired plasma (Figure 5A). The cluster heatmap shows the up‐ and down‐regulated relationships of partial differentially abundant metabolites between cystic fluid and paired plasma (Figure 5B). Notably, choline was significantly enriched in the cystic fluid, whereas betaine and lysoPC contents were downregulated in the cystic fluid. In the human body, betaine is an oxidative metabolite of choline, which undergoes oxidative removal of its methyl group to form betaine. At the same time, betaine also participates in the methyl cycle and methylation reactions as a more efficient methyl donor in vivo, indirectly reducing the consumption of choline as a methyl donor, thereby “saving” choline and increasing its availability for other physiological functions, such as PC synthesis [36]. LysoPC is a hydrolysate of PC that can be further hydrolyzed to choline. The choline produced by LysoPC hydrolysis can in turn be used to synthesize PC via the Kennedy pathway. The “PC‐LysoPC‐Choline‐PC” cycle involves functional coupling during biomembrane renewal and lipid transport.

To quantitatively verify the metabolite content differences between cystic fluid and paired plasma, targeted metabolomics was performed. A total of 344 metabolites were detected via targeted metabolomics and divided into 9 categories according to HMDB superclass classification (Figure 5C). In superclass 6, the contents of choline, ethanolamine and trimethylamine (a metabolite of choline) were increased in the cystic fluid (no significant difference was found in the ethanolamine content). Lollipop charts of differential metabolism and targeted quantitative results by LC‐MS/MS also revealed that choline was significantly upregulated in the cystic fluid (Figure 5D,E). In addition, we also detected abundant fatty acids enriched in cystic fluid (Figure 5D), which is consistent with the oil‐like properties of ACP cystic fluid observed clinically.

The above results collectively revealed a choline‐enriched cystic fluid microenvironment in ACP. Notably, we also found higher expression of the choline transporter SLC44A1 in ACP tumor tissues than in paratumor tissues and in recurrent tissues than in primary tissues (Figure 5F). These results coincided with the gene expression of choline transporters in the single‐cell spatial transcriptomics data (Figure 4E,F); it is reasonable to infer that ACP tumor cells continuously take in choline/ethanolamine from cystic fluid to maintain PC synthesis.

### 2.6 Autophagy Pathway Activation in ACP Tumor Regions and Recurrent Tumor Epithelium Tissues

In the aforementioned investigation, we demonstrated the remodeling and activation of the PC and PE synthesis pathways in ACP tumor regions and recurrent tumor epithelium tissues via multiomics approaches. PC and PE, the two most abundant glycerophospholipids in the cell membrane, jointly maintain numerous physiological activities of cells. Mediating cell autophagy is an important physiological function of PC and PE. Several studies have shown that PC and PE can positively regulate autophagy processing [37, 38]. Hence, we speculated that the enrichment of PC and PE would impact the autophagic function of ACP.

**FIGURE 6:**
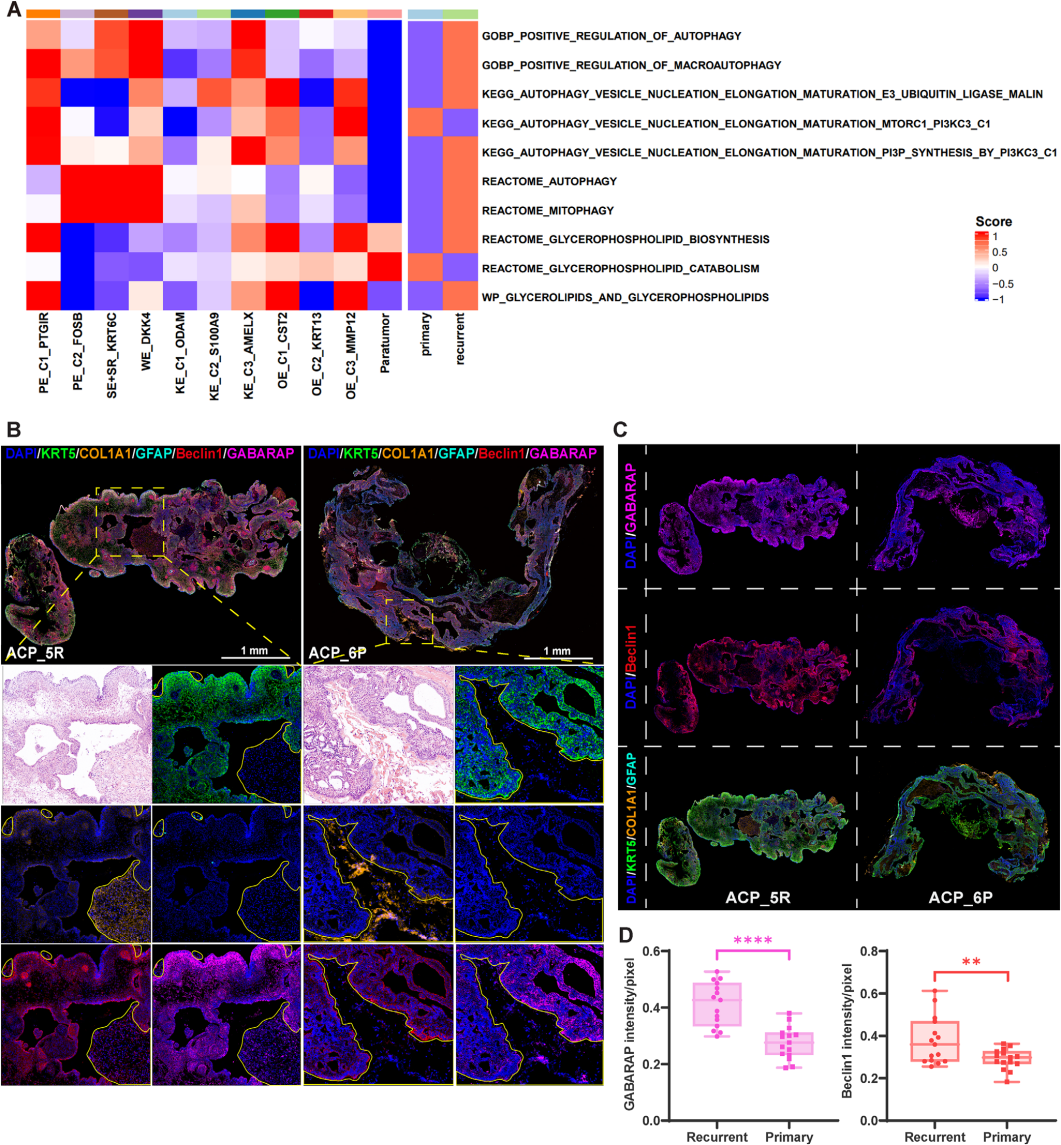
Verification of autophagy pathway activation features in ACP. (A) Heatmaps showing the GSVA scores of autophagy‐ and glycerophospholipid‐related pathways between tumor epithelial cell subpopulations and paratumor tissues or between primary and recurrent tumor epithelial cells. (B, C) Adjacent sections of ones performing single‐cell spatial transcriptomics were used for multi‐IHC staining, and the expression levels of KRT5, COL1A1, GFAP, Beclin1, and GABARAP were explored. ACP_5R and ACP_6P tissues are shown as representatives; scale bar = 1 mm. B: Differential expression of the autophagy markers Beclin1 and GABARAP between tumor tissues and paratumor tissues. The yellow lines mark the paratumor regions. C: Differential expression of the autophagy markers Beclin1 and GABARAP between recurrent and primary tumor epithelial tissues. (D) Statistical analysis of GABARAP and Beclin1 expression intensity between recurrent and primary tumor epithelium tissues (*n* = 15). *p* values were calculated via the Student's *t* test. ****p < 0.0001, **p < 0.01.

To test this hypothesis, GSVA scoring of autophagy‐ and glycerophospholipid‐related pathways was performed between different tumor epithelial cell subpopulations and paratumor cells or between primary and recurrent tumor epithelial cells. The scoring results in Figure 6A revealed that autophagy‐related pathways in several databases, such as positive regulation of autophagy (GOBP), autophagy vesicle nucleation elongation maturation (KEGG) and mitophagy (REACTOME), were upregulated through tumor epithelial cell subpopulations to varying degrees, whereas the expression levels of these pathways were significantly lower in paratumor cells. These pathways were also upregulated in recurrent tumor epithelial cells. Correspondingly, the glycerophospholipid biosynthesis and catabolism pathways were upregulated and downregulated, respectively, in tumor epithelial cell subpopulations/recurrent tumor epithelial cells. Among the tumor epithelial cell subpopulations, the PE_C1_PTGIR, KE_C3_AMELX, OE_C1_CST2, and OE_C3_MMP12 clusters exhibited preferable correspondence between glycerophospholipid hypersynthesis and autophagy pathway activation.

In addition, multi‐IHC was performed to explore the expressions of KRT5, COL1A1, GFAP, Beclin1, and GABARAP in 10 ACP tissues (adjacent tissues used for single‐cell spatial transcriptomics analysis) (Figures 6B,C and S8). KRT5 (a marker of ACP tumor epithelial cells) was used to localize to tumor regions, and COL1A1 (a marker of CAFs and fibroblasts) and GFAP (a marker of astrocytes) were utilized to localize to paratumor regions. Beclin1 and GABARAP play indispensable roles in autophagy, which involves autophagy initiation and autophagosome membrane elongation and maturation [39, 40, 41]. As shown in Figures 6B and S8, Beclin1 and GABARAP were more highly expressed in tumor tissues than in paratumor tissues. Moreover, as shown in Figures 6C,D and S8, increased Beclin1 and GABARAP expressions were observed in recurrent tumor epithelial cells.

In summary, consistent with the activation features of the PC and PE synthesis pathways, autophagy pathway activation was verified in ACP tumor regions and recurrent tumor epithelium tissues.

## 3 Conclusion

The key challenges of ACP in clinical practice are the difficulty of surgery and severe postoperative complications caused by the invasive growth of tumors, the high recurrence rate, and the limitations of existing treatment methods. However, the heterogeneity and structural complexity of ACP also present challenges to the exploration of tumor development and recurrence mechanisms and the discovery of therapeutic targets.

In this study, we pioneered the integration of spatially resolved multiomics, including single‐cell spatial transcriptomics and spatially resolved metabolomics, complemented with bulk metabolomics (untargeted/targeted), to dissect the molecular and metabolic architecture of ACP and decipher ACP progression and recurrence mechanisms. By leveraging single‐cell resolution mapping and in situ metabolic profiling, this approach overcomes the limitations of bulk tissue analysis and enables unprecedented insights into the heterogeneous TME. First, spatial cellular heterogeneity was observed, including the identification of 10 distinct tumor epithelial subpopulations and their diverse transcriptional signatures. Second, metabolic remodeling was clarified, as evidenced by PC/PE synthetic hyperactivity within tumor epithelial cells tightly coupled with choline/ethanolamine uptake via the SLC44A1 transporter from cystic fluid. Third, functional coupling suggested that glycerophospholipid metabolic remodeling directly drove autophagy activation in tumor epithelial cells. In addition, the activation of the above metabolic and autophagy pathways is more significant in recurrent ACP compared with primary ones.

Compared with previous single‐cell transcriptomics and spatial transcriptomics studies of ACP [11, 12], the single‐cell spatial transcriptomics applied in this study—based on its single‐cell resolution and integration of spatial information—allows us to obtain a more accurate and precise understanding of the transcriptional characteristics of ACP. In addition, the inclusion of paired primary and recurrent samples allowed us to further explore the mechanism of ACP recurrence at the transcriptional level. Ten cell populations were annotated in our study, and the cell composition aligned with previous studies and included mainly tumor epithelial cells, stromal cells, immune cells, and glial cells [21, 22]. However, although abundant macrophages and B cells were detected in the ACP TME, T cells and other immune cells were not annotated (Figure S2), possibly because of the inherently low abundance of these immune cells in the ACP TME [13, 22] and the degradation of low‐abundance mRNAs in the FFPE samples. In addition, the spatial colocalization features of different cell populations were elucidated in our study. We found that macrophages often colocalized with astrocytes, which was consistent with the immunological infiltration in the glial‐reactive layers outside the ACP revealed in previous studies [22]. Macrophages (especially SPP1+ macrophages) and B cells also frequently encircle keratin nodules; this phenomenon has also been mentioned in previous studies, which suggested that cell necrosis during keratin nodule formation could attract immune cell infiltration and induce nonspecific inflammation [42]. Notably, the macrophages in this study were divided into SPP1‐positive and SPP1‐negative populations. Several studies have revealed that the expression of SPP1 in macrophages is significantly correlated with the occurrence and prognosis of various diseases, especially cancers other than ACP [43, 44, 45]. SPP1+ macrophages are often enriched in hypoxic and necrotic tumor regions and are associated with poor outcomes in cancer patients [45], which aligns with our findings described above.

Several studies have investigated tumor epithelial heterogeneity within ACP [13, 21], and our study was conducted from a more detailed single‐cell spatial perspective. By reclustering tumor epithelial cells, we obtained 10 subpopulations with significant transcriptional differences. Consistent with previous studies, our study showed that the WE structure (WE_DKK4) manifested as WNT pathway activation. Furthermore, our study revealed many uncharacterized transcriptional signatures of tumor epithelial cell populations. For example, although the PE_C1_PTGIR and PE_C2_FOSB clusters were localized in the PE structure, their transcriptional signatures were associated with immune regulation and cell proliferation, respectively. These findings also suggest the important role of the inflammatory microenvironment in ACP tumor progression. The three clusters of cells—KE_C1_ODAM, KE_C2_S100A9, and KE_C3_AMELX—were located in the wet keratin structure. Interestingly, energy metabolism and cholesterol metabolism pathways were significantly enriched in these clusters. This finding demonstrates the specificity of the ACP metabolic microenvironment and implies that the abundant cholesterol in ACP tumors and cystic fluid may be secreted by KE cells [46, 47]. In addition, although no significant difference was observed in the proportion of the main cell population in the primary and recurrent samples (data not shown), the proportions of the KE_C2_S100A9 and OE_C2_KRT13 subpopulations were significantly greater in the recurrent samples. Consistent with our previous findings, a greater presence of wet keratin structures was observed in recurrent cases, which suggests that the unique metabolic characteristics of KE cells may play an important role in the recurrence of ACP.

By further integrating single‐cell spatial transcriptomics data with spatially resolved metabolomics data, we revealed significant activation of PC and PE synthetic pathways in ACP tumor regions compared with paratumor regions, as well as in recurrent tumor epithelium tissues compared with primary tumor epithelium tissues. This phenomenon is closely related to the proliferation demand of tumor cells. As the main components of the cell membrane, the synthetic hyperactivity of PC and PE could support the rapid growth and invasion of tumor cells [48, 49]. Notably, the significant enrichment of choline in cystic fluid may provide raw material for PC synthesis, and tumor cells increase their uptake capacity by upregulating the expression of the choline/ethanolamine transporter SLC44A1 [50, 51], which results in “cystic fluid–tumor cell” metabolic coupling.

Another important finding in the present study was that autophagy pathway activation was observed in ACP tumor regions (and recurrent tumor epithelial tissues). We hypothesized that autophagy pathway activation was the downstream consequence of excessive synthesis of PC and PE. PC and PE play crucial roles in the autophagy process [37, 38]. PC provides the building blocks for autophagosome biogenesis. Its hydrolysis by phospholipases generates lipid mediators that can modulate autophagy signaling pathways, such as activating protein kinases involved in autophagosome formation [52, 53]. Moreover, PE is essential for the expansion and completion of autophagosomes. Specifically, cytosolic microtubule‐associated protein 1 light chain 3 (LC3) undergoes posttranslational modification, where its C‐terminus is conjugated to PE, enabling LC3 to anchor onto autophagosome membranes. This LC3–PE complex facilitates the recognition and engulfment of damaged organelles and misfolded proteins [54, 55]. In this study, Beclin1 and GABARAP were used as autophagy markers for multiple IHC validations of ACP tissues. Beclin1 acts as a core component of the class III phosphatidylinositol 3‐kinase (PI3KC3) complex. This complex generates phosphatidylinositol 3‐phosphate (PI3P), which is a lipid signal critical for initial membrane structure formation and the expansion of autophagosomes [39, 40]. GABARAP, alongside LC3, belongs to the same ubiquitin‐like protein family. GABARAP also undergoes posttranslational modification by PE, and the GABARAP–PE complex anchors to autophagosome membranes and mediates multiple functions, such as membrane elongation and maturation [41].

Autophagy, a conserved cellular degradation process, plays a dual role in tumorigenesis and cancer progression [56]. As a tumor suppressor, autophagy eliminates damaged organelles, misfolded proteins, and reactive oxygen species, thereby preventing genomic instability and inhibiting oncogenic transformation. Conversely, in established and metastasizing tumors, autophagy supports cancer cell survival under metabolic stress (such as nutrient deprivation and hypoxia) by recycling macromolecules for energy production and maintaining proteostasis. This adaptive autophagy also facilitates tumor cell invasion and metastasis by modulating the extracellular matrix and enabling evasion of immune surveillance. In addition, autophagy can confer resistance to chemotherapy and targeted therapies by protecting cancer cells from drug‐induced apoptosis.

On the basis of the discovery of the accumulation of choline in cystic fluid and its metabolic coupling to the PC/PE synthesis‒autophagy axis in ACP, this study provides a rational framework for the development of novel therapeutic strategies: 1. SLC44A1 inhibition: Targeting the choline/ethanolamine transporter could disrupt the “cystic fluid–tumor cell” metabolic coupling and starve cells of the substrate, subsequently suppressing PC/PE hyperproduction. Small molecule inhibitors may selectively block SLC44A1, synergizing with existing therapies [57]; 2. PC/PE synthesis interference: Pharmacological inhibition of key enzymes in the Kennedy pathway (e.g., CEPT1 and CHPT1); 3. Metabolite‒drug conjugates (MDCs): Leveraging the ability of ACP to take up choline, a prodrug strategy could conjugate cytotoxic anticancer drugs (e.g., PTX and doxorubicin) to choline analogs. Upon uptake via SLC44A1, the conjugate linker is cleaved by a tumor‐specific enzyme, enabling tumor‐specific drug release while protecting healthy tissues—a mechanism validated in recent studies on metabolic vulnerability and spatially resolved drug conjugate development [58]. This approach synergizes with ACP's unique metabolic dependencies, offering the dual advantages of enhanced tumor targeting (via choline gradient‐guided accumulation) and reduced systemic toxicity (via spatially restricted activation).

In conclusion, through multiomics integration analysis, this study systematically elucidated the tumor heterogeneity and tumor epithelial diversity in ACP; meanwhile, it revealed the newly found “cystic fluid–tumor cell” metabolic coupling that mediates active choline/ethanolamine uptake of tumor cells and PC and PE synthesis pathway remodeling that mediates autophagy pathway activation within ACP. In addition, the activation of the above metabolic and autophagy pathways is more significant in recurrent ACP compared with primary ones. Together, these findings lay a critical foundation for elucidating the pathogenesis of ACP, managing tumor recurrence, and designing novel therapeutic strategies.

However, this study also has several limitations. First, the sample size was relatively small because of the rarity of ACP, which may affect the accuracy of the conclusions. Second, the spatial metabolic heterogeneity has not been elaborately elucidated at the tumor epithelial cell subpopulations level. Third, the present study is “observational” only. Future research directions may include expanding the sample size and conducting long‐term follow‐up of patients; the metabolic features of different tumor epithelial cell subpopulations need to be further integrated and analyzed with their transcriptomic information to clarify the interactions and evolution among them; moreover, more explorations are needed to verify the functional role of PC/PE synthesis and the autophagy pathway in ACP via molecular biology approaches.

## 4 Experimental Section

### 4.1 Clinical Sample Collection and Ethics Approval

All 24 patients enrolled in this study underwent surgery at Sanbo Brain Hospital, Capital Medical University, and received pathological confirmation of primary or recurrent ACP between 2017 and 2024. The sample included 17 males and 7 females, with ages ranging from 1 to 57 years. Formalin‐fixed paraffin‐embedded (FFPE) tumor tissue samples (including 4 paired primary and recurrent samples whose duration of recurrence ranged from 2 to 6 years), frozen fresh tumor tissue samples, cystic fluid samples (paired with plasma samples) were collected for single‐cell spatial transcriptome sequencing, spatially resolved metabolomics analysis, and bulk metabolomics analysis, respectively. None of the participants had received radiotherapy, chemotherapy, or any other form of antitumor therapy within one year prior to surgery. The patient clinical information and sample information are summarized in Tables S1 and S2.

This study complies with all relevant ethical regulations. This study was approved by the Institutional Review Board of Sanbo Brain Hospital (approval no. SBNK‐YJYS‐2024‐002‐01), and informed consent was obtained from all participants, their parents, or legal guardians. All procedures were performed in accordance with the guidelines of the Institutional Review Board of Sanbo Brain Hospital and the Declaration of Helsinki.

### 4.2 Single‐Cell Spatial Transcriptome Sequencing

The CosMx SMI platform (6K mRNA panel; NanoString Technologies, Washington, DC, USA, RRID: SCR_023909) was used for single‐cell spatial transcriptome sequencing.

#### 4.2.1 Sample Preparation

Ten ACP FFPE samples were collected for single‐cell spatial transcriptome sequencing (Table S1), including 4 paired primary and recurrent samples (paired samples from the same patient), 1 primary sample, and 1 recurrent sample (unpaired). After quality inspection, FFPE tissue sections (5 µm) were mounted onto SuperFrost Plus slip‐proof slides (VWR, Pennsylvania, USA) within the detection area of CosMx SMI (20 mm × 15 mm). Adjacent sections were stained with H&E and evaluated by pathologists to distinguish pathological structures. FFPE tissue sections were prepared for CosMx SMI profiling as described in reference [28].

#### 4.2.2 CosMx SMI Instrument Run

The RNA target readout on the CosMx SMI instrument was performed as described previously [27, 28]. The slides of ACP FFPE sections were loaded onto a CosMx SMI instrument. The slides were stained with specific antibodies (anti‐pan‐CK, anti‐CD298, and anti‐CD45) and DAPI for visualization of cell morphology, and the field of view (FOV) was selected. A total of 386 FOVs were placed on the tissue to match the regions of interest from adjacent sections. After FOV selection, the slides were hybridized with 6,000 human mRNA‐targeting probes. The assembled flow cell was loaded onto the instrument, and reporter wash buffer was flowed to remove air bubbles. Probe hybridization began by flowing reporter pool 1 into the flow cell and incubating for 15 min. Then, the unbound probes were washed away with reporter wash buffer, and imaging buffer was added to the slides prior to imaging. The photocleavable linkers on the fluorophores of the reporter probes were then released by UV illumination, followed by washing with strip wash buffer. The fluorescent barcodes were subsequently read by a CosMx imager and decoded by probe identity to generate in situ CosMx probe detection data. The three immunofluorescent antibodies and DAPI were used to segment the tissues into cells via the machine learning algorithm CellPose. Target transcripts were assigned to cells and subcellular compartments on the basis of segmentation boundaries.

#### 4.2.3 Data Processing

Following the generation of the data, Seurat (v5.0.3, RRID: SCR_007322), scanpy (v1.9.8, RRID: SCR_018139), and Giotto (v4.0.5, RRID: SCR_027369) were used for downstream analysis. In brief, quality control across multiple dimensions was first conducted, following Nanostring official documentation, to filter out low‐quality cells. Next, scanpy was used to perform dimensionality reduction and clustering. Here, the Leiden algorithm was chosen for graph‐based clustering. The processed data were then imported into R for further analysis. Using the “InsituType” [59] and “FindMarkers” [60] functions, the cells were annotated with the appropriate reference dataset. InSituCor was used to identify spatial co‐expressed gene modules. GSVA was performed to evaluate pathway activity at the single‐cell level via GSVA (v1.50.5, RRID: SCR_021058). The analysis was conducted via predefined gene sets derived primarily from public databases such as GO (RRID: SCR_002811) and KEGG (RRID: SCR_012773). Following GSVA, the enrichment scores were averaged across cells of the same cell type to facilitate downstream visualization.

### 4.3 Spatially Resolved Metabolomics Analysis

The air flow‐assisted desorption electrospray ionization integrated mass spectrometry imaging (AFADESI‐MSI) platform was used for spatially resolved metabolomics analysis as described previously [31].

#### 4.3.1 Sample Preparation

Ten ACP tissues (5 primary ACPs and 5 recurrent ACPs) were collected for spatially resolved metabolomics analysis (Table S1). The surgically removed ACP tissues were rapidly frozen in liquid nitrogen, fixed through OCT embedding adhesive (Leica, Wetzlar, Germany), and then prepared into 12 µm cryosections on positively charged anti‐fade glass slides (Thermo Scientific, Massachusetts, USA). The tissue sections were vacuum‐dried for 1 h at −20°C and room temperature before mass spectrometry imaging. The adjacent sections were subjected to H&E staining and evaluated by pathologists to distinguish the pathological structures.

#### 4.3.2 AFADESI‐MSI Instrument Run

An Orbitrap Explores 120 high‐resolution mass spectrometer (Thermo Scientific, Massachusetts, USA, RRID: SCR_027370) equipment with a custom‐built AFADESI‐MSI platform was used to collect data in both positive‐ and negative‐ion mode separately, in the mass range of m/z 70–1000. The mass accuracy was ensured by standard sample calibration before the experiment (error < 5 ppm). The spray solvent was acetonitrile and water (8:2, v/v), and the flow rate of the spray solvent was set to 5.0 µL min^−1^. The sprayer voltages were set at 7 and −7 kV in positive‐ and negative‐ion modes, respectively. The extraction gas flow rate of the AFADESI ion source was 40 L min^−1^. The flow rate of nebulizing gas was set to 0.7 MPa. Imaging analysis was performed by continuously scanning the tissue section in the *x*‐direction at 0.15 mm s^−1^, separated by a 0.2 mm vertical step in the *y*‐direction.

#### 4.3.3 Mass Spectrometry Imaging Data Processing

The raw mass spectrometry imaging data were converted to .CDF format files via Xcalibur software (Thermo Scientific, Massachusetts, USA, RRID: SCR_014593). MassImager Pro software (Chemmind Technologies, Beijing, China) was used for background subtraction, image reconstruction, ROI selection, and data extraction. The extraction data (m/z intensity) of each ROI were subsequently imported into MarkerView 1.2.1 (ABSCIEX, Boston, USA, RRID: SCR_027371) for peak picking, peak alignment, and isotope ion deletion. Finally, the preprocessed data were exported for subsequent statistical analysis. The metabolite annotation was conducted on the basis of the HMDB (RRID: SCR_007712) and METLIN databases (RRID: SCR_010500) (mass error < 5 ppm). The processed data were subjected to multivariate data analysis, including Pareto‐scaled PCA and OPLS‐DA. A VIP value > 1.0 in the OPLS‐DA was screened as a differentially abundant metabolite. Statistical analysis was performed via the Mann‒Whitney *U* test, with a significance threshold of *p* < 0.05. Pathway enrichment analysis was performed via the MetaboAnalyst (RRID: SCR_015539) and KEGG databases.

### 4.4 Untargeted and Targeted Metabolomics Analysis

#### 4.4.1 Sample Preparation

Eleven cystic fluid samples and 9 paired plasma samples were collected for untargeted metabolomics analysis, and 6 cystic fluid samples and 6 paired plasma samples were collected for targeted metabolomics analysis (Table S2). To remove the protein and extract the metabolites, the samples were added with 400 µL of cold methanol/acetonitrile (1:1, v/v) extraction solvent and then adequately vortexed. For targeted metabolomics analysis, for absolute quantification of the metabolites, stock solutions of stable‐isotope internal standards (Sigma‐Aldrich, Darmstadt, Germany) were added to the extraction solvent simultaneously. The mixture was collected into a new centrifuge tube and centrifuged at 14,000× *g* for 20 min at 4°C to collect the supernatant. The supernatant was dried in a vacuum centrifuge, redissolved in 100 µL of acetonitrile/water (1:1, v/v) and centrifuged at 14,000× *g* at 4°C for 15 min. Then, the supernatant was injected into the LC‐MS/MS system.

#### 4.4.2 Untargeted Metabolomics Analysis

UHPLC‐MS/MS analyses were performed via a Vanquish UHPLC system (Thermo Scientific, Massachusetts, USA, RRID: SCR_025713) coupled with an Orbitrap Q Exactive HF mass spectrometer (Thermo Scientific, Massachusetts, USA, RRID: SCR_020425). The samples were injected onto a Hypersil Gold column (100 × 2.1 mm, 1.9 µm) via a 12‐min linear gradient at a flow rate of 0.2 mL min^−1^. The Q Exactive HF mass spectrometer was operated in positive/negative polarity mode with a spray voltage of 3.5 kV, capillary temperature of 320°C, sheath gas flow rate of 35 psi, aux gas flow rate of 10 L min^−1^, S‐lens RF level of 60, and aux gas heater temperature of 350°C.

#### 4.4.3 Targeted Metabolomics Analysis

Analyses were performed via a 1290 Infinity UHPLC system (Agilent Technologies, California, USA, RRID: SCR_019378) coupled with a 6500+ QTRAP mass spectrometer (AB Sciex, Boston, USA, RRID: SCR_021831). The analytes were separated on HILIC (Waters UPLC BEH amide column, 2.1 mm × 100 mm, 1.7 µm) and C18 columns (Waters UPLC BEH C18‐2.1×100 mm, 1.7 µm). A 6500+ QTRAP was applied in positive and negative switch mode. The MRM method was used for mass spectrometry quantitative data acquisition. Polled quality control (QC) samples were set in the sample queue to evaluate the stability and repeatability of the system.

#### 4.4.4 Data Processing

The raw data files generated by UHPLC‐MS/MS were reprocessed, including peak alignment, peak picking, annotation, and quantitation for each metabolite. The processed data were subjected to multivariate data analysis, including PCA and OPLS‐DA. Statistical analysis was performed via the Mann‒Whitney *U* test to screen significantly changed metabolites, with a significance threshold of *p* < 0.05.

### 4.5 Multiplex Immunohistochemistry

The expressions of KRT5, COL1A1, GFAP, Beclin1, and GABARAP in ACP were explored. Adjacent sections of ones performing single‐cell spatial transcriptomics were used for multiplex immunohistochemical (multi‐IHC) staining. The FFPE sections were baked at 65°C for 2 h and then dewaxed and hydrated with xylene and gradient alcohol. EDTA buffer (pH 9.0) or sodium citrate buffer (pH 6.0) was used for antigen retrieval. The sections were preincubated with 10% goat serum to block nonspecific binding sites. A multi‐IHC staining kit (Baiqiandu Biotechnology, Wuhan, China) was used according to the manufacturer's instructions. The slides were incubated with the primary antibody and then with the secondary antibody, and tyrosine signal amplification was performed. The sections were subsequently subjected to microwave heat repair, and the next round of staining was performed. After all the indicators were stained, DAPI was used to mark the nucleus. Pannoramic SCAN and 3DHISTECH CaseViewer (3DHISTECH, Budapest, Hungary) were used for multispectral slice scanning and image analysis. All antibody and fluorescence marker information is included in Table S3, the primary antibodies used were as follows: rabbit anti‐KRT5 (CST, cat# 71536, RRID:AB_3101753), rabbit anti‐COL1A1 (CST, cat# 72026, RRID:AB_2904565), mouse anti‐GFAP (CST, cat# 3670, RRID:AB_561049), rabbit anti‐Beclin1 (Abcam, cat# ab210498, RRID:AB_2810879), and rabbit anti‐GABARAP (Abcam, cat# ab109364, RRID:AB_10861928).

### 4.6 Hematoxylin and Eosin Staining

Adjacent sections of ones performing single‐cell spatial transcriptomics and spatially resolved metabolomics were used for H&E staining. The tissue sections were incubated at 65°C for 2 h, deparaffinized with xylene, and rehydrated with an ethanol gradient (100%–50%). Next, the sections were stained with hematoxylin solution for 5 min, dipped in 1% acid ethanol (1% HCl in 70% ethanol), and then rinsed with distilled water. Then, the sections were stained with eosin solution for 3 min, dehydrated with ethanol, and cleared in xylene.

### 4.7 Western Blotting

Proteins were extracted with RIPA lysis buffer containing protease inhibitors and phosphatase inhibitors (CWbio, Jiangsu, China), and their concentrations were measured with a BCA protein assay kit (Solarbio, Beijing, China). Following separation by SDS‒polyacrylamide gel electrophoresis, the proteins were transferred onto PVDF membranes (Millipore, Massachusetts, USA) via the Bio‐Rad Mini Trans‐Blot System. Then, the membranes were blocked for 1 h with 5% nonfat milk in TBST buffer at room temperature and incubated with primary antibody solutions at 4°C overnight. The blots were washed with TBST and subsequently incubated with HRP‐conjugated secondary antibodies at room temperature for 1 h. After being washed with TBST three times, the blots were incubated in ECL sensitivity luminescent solution (Applygen, Beijing, China) and imaged. The primary antibodies used were as follows: rabbit anti‐SLC44A1 (Invitrogen, cat# PA5‐102034, RRID: AB_2851465) and mouse anti‐β‐Tubulin (CST, cat# 86298, RRID:AB_2715541).

### 4.8 Statistical Analysis

Data statistics were performed based on GraphPad Prism 8.0 software (RRID:SCR_002798). The *p* value of the difference between different groups was obtained by performing Mann–Whitney *U* test, Student's *t*‐test, and one‐way analysis of variance (ANOVA). When *p* < 0.05, differences were considered statistically significant.

## Funding

Chinese Academy of Medical Sciences (CAMS) Innovation fund for Medical Science (Grant number: 2021‐I2M‐1‐057); The National Natural Science Foundation of China (Grant number: 21927808).

## Conflicts of Interest

The authors declare no conflicts of interest.

## Supporting information




**Supporting File**: advs73640‐sup‐0001‐SuppMat.docx.

## Data Availability

The data will be made available upon reasonable request. Correspondence and request for materials should be addressed to Xin Li, Fangjun Liu, and Yan Li.
