# Supplementary material for: Spatially Resolved Multiomics Reveals Metabolic Remodeling and Autophagy Activation in Adamantinomatous Craniopharyngiomas
Source: Adv Sci (Weinh). 2026 Jan 5;13(17):e16965. doi: 10.1002/advs.202516965 (PMC13042386; doi:10.1002/advs.202516965)
Supplement: Supplementary file 1 — Supporting File: advs73640‐sup‐0001‐SuppMat.docx. [file ADVS-13-e16965-s001.docx]

Supporting Information

Spatially Resolved Multiomics Reveals Metabolic Remodeling and Autophagy Activation in Adamantinomatous Craniopharyngiomas


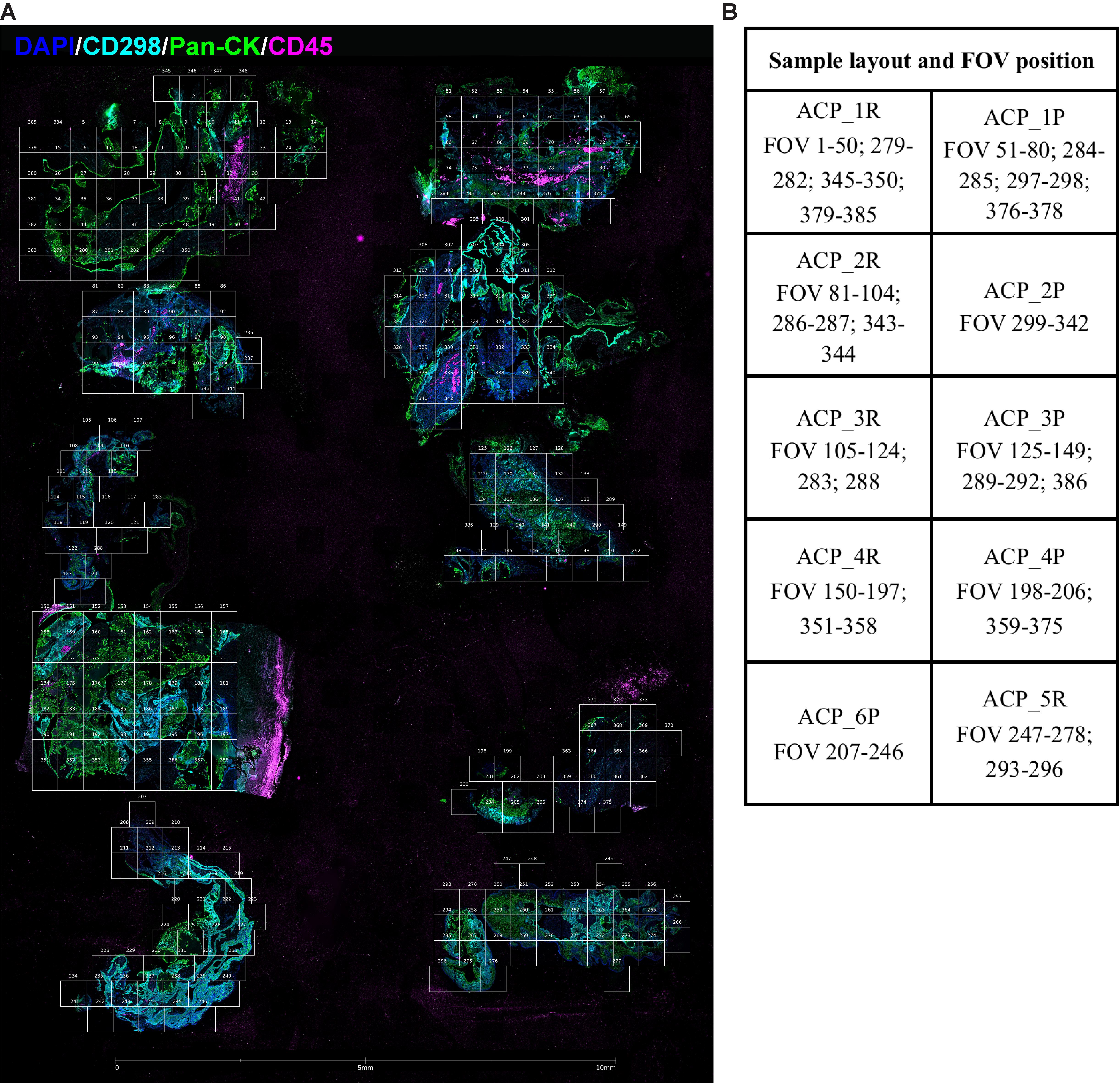
*Dongting Chen, Yahui Gao, Yulin Wang, Ting Lei, Zheng Qu, Yuhan An, Jiaxu Fu, Xin Li*, Fangjun Liu* and Yan Li**

Supplementary Figure. S1 The immunofluorescence images of morphological marker used for single-cell recognition and segmentation (A), and the FOV positions (B). DAPI, CD298, Pan-CK and CD45 are used to mark cell nuclear, cell membrane, epithelial cells and leukocytes, respectively.


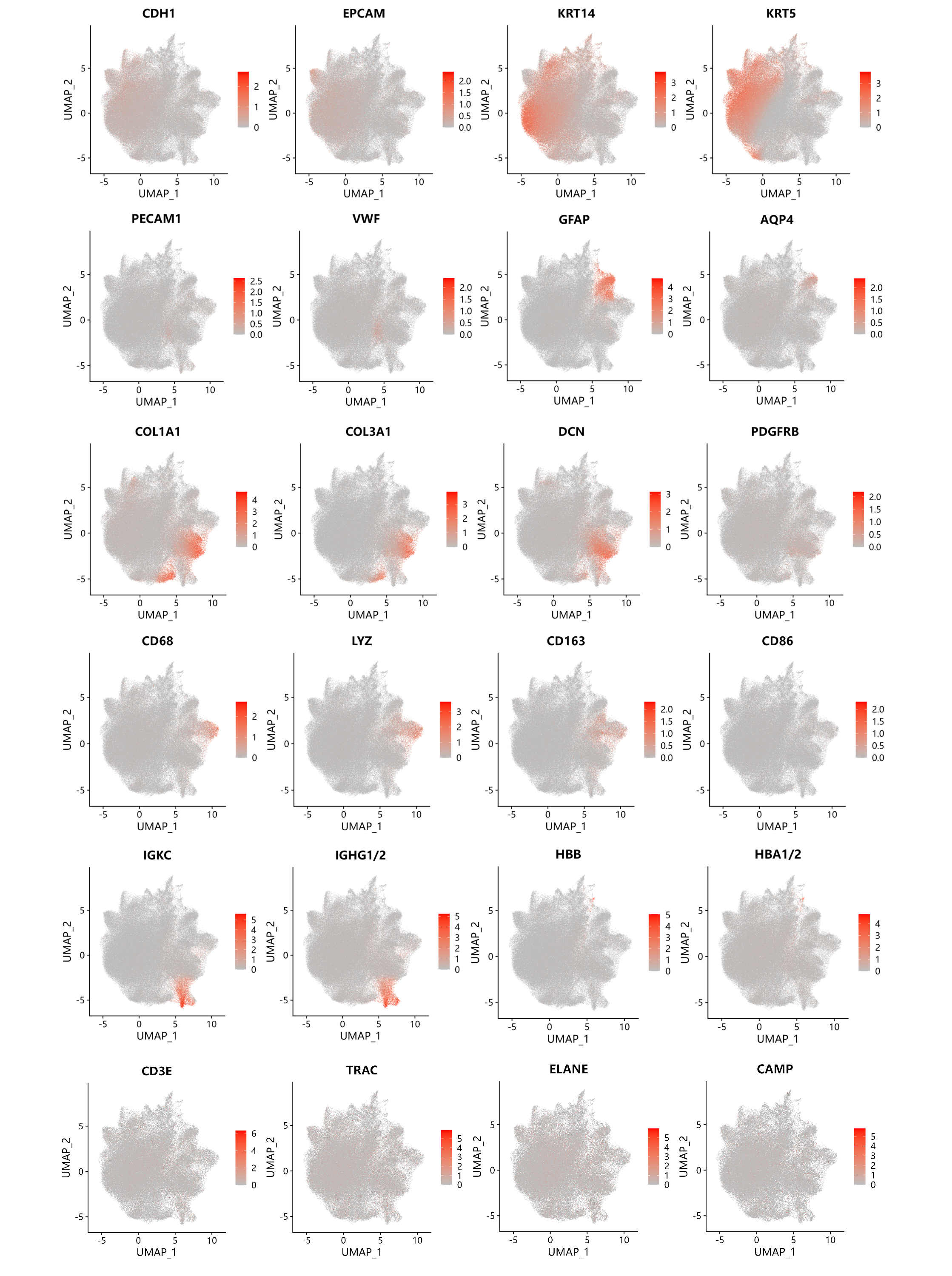
 **Supplementary Figure. S2 Feature plots depicting the activity and distribution of marker genes for each cell population.** CDH1, EPCAM, KRT14, and KRT5 for epithelial cells; PECAM1 and VWF for endothelial cells; GFAP and AQP4 for astrocytes; COL1A1, COL3A1, DCN, and PDGFRB for tumor-associated fibroblasts (CAFs) and fibroblasts; CD68, LYZ, CD163 and CD86 for SPP1 - and SPP1 + macrophages; IGKC and IGHG1/2 for B cells; HBB and HBA1/2 for red blood cells (RBC); CD3E and TRAC for T cells; ELANE and CAMP for neutrophils.


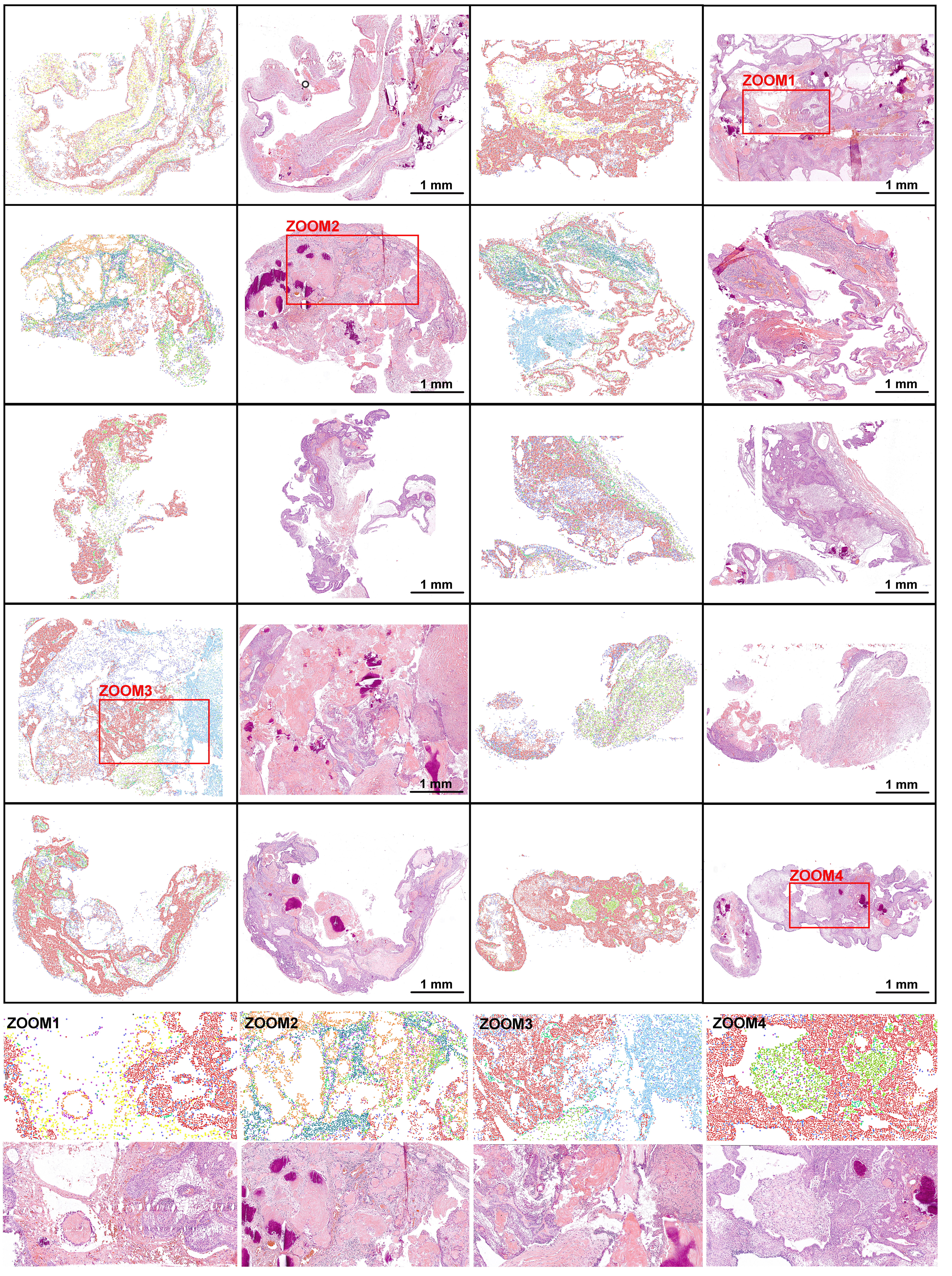
Supplementary Figure. S3 The spatial distribution of single cell populations corresponding to the pathological structures in the H&E-stained adjacent slides. Each dot represents a cell, and the legend (colors representing cell types) is the same as in Figure 1C.

Supporting Information

Spatially Resolved Multiomics Reveals Metabolic Remodeling and Autophagy Activation in Adamantinomatous Craniopharyngiomas

*Dongting Chen, Yahui Gao, Yulin Wang, Ting Lei, Zheng Qu, Yuhan An, Jiaxu Fu, Xin Li*, Fangjun Liu* and Yan Li**

**
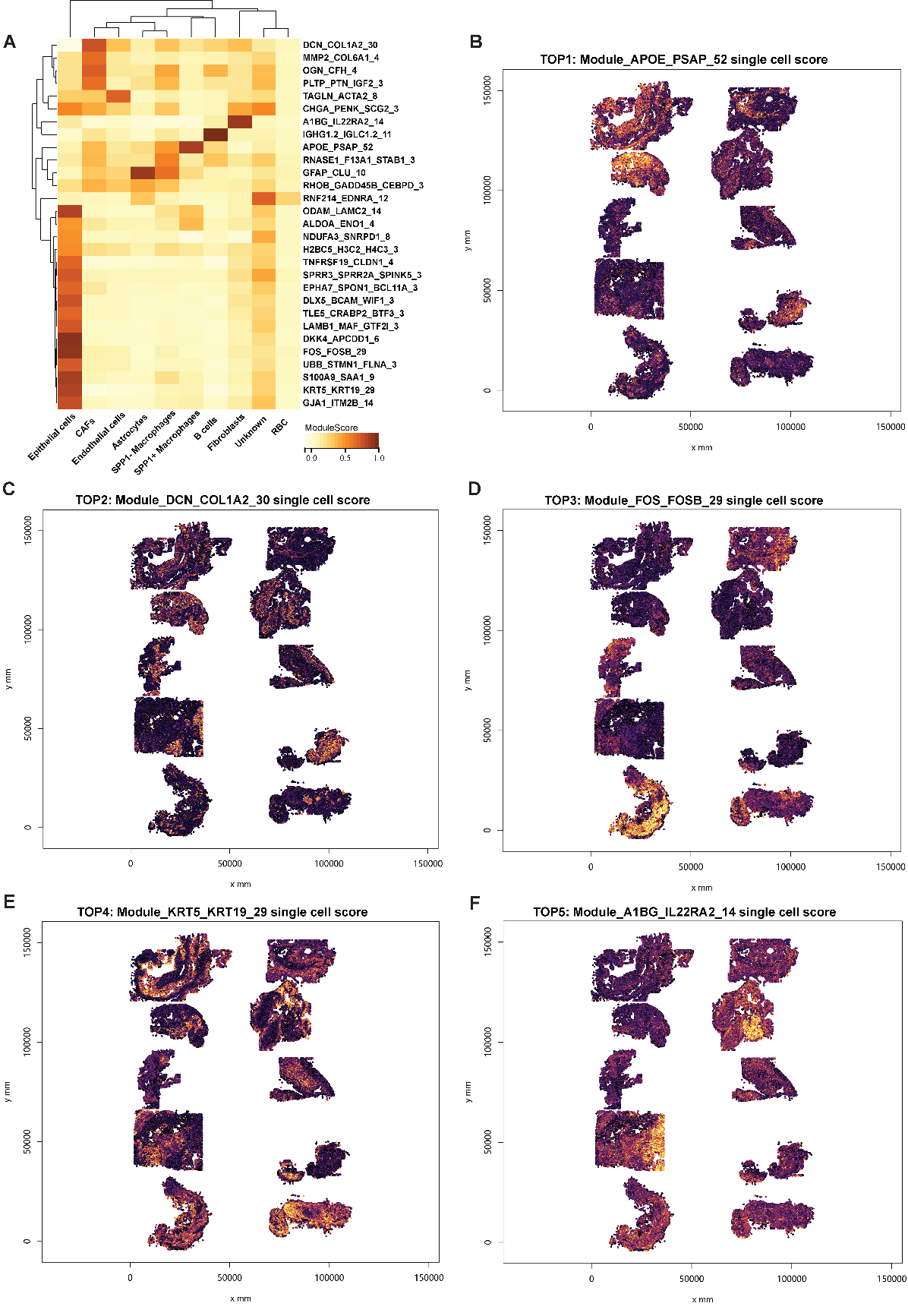
**

**Supplementary Figure. S4 Spatially co-expressed gene module analysis in ACP.** (A) Heatmap showing module score values for each co-expressed gene module in cell subgroups. Modules are named using their 2-3 most influential genes, and the number of genes. E.g., the module APOE_PSAP_52 is most impacted by APOE and PSAP, and it has 52 total genes. (B-F) Spatial in-situ heatmaps of the top 5 modules (ranked by number of genes included).


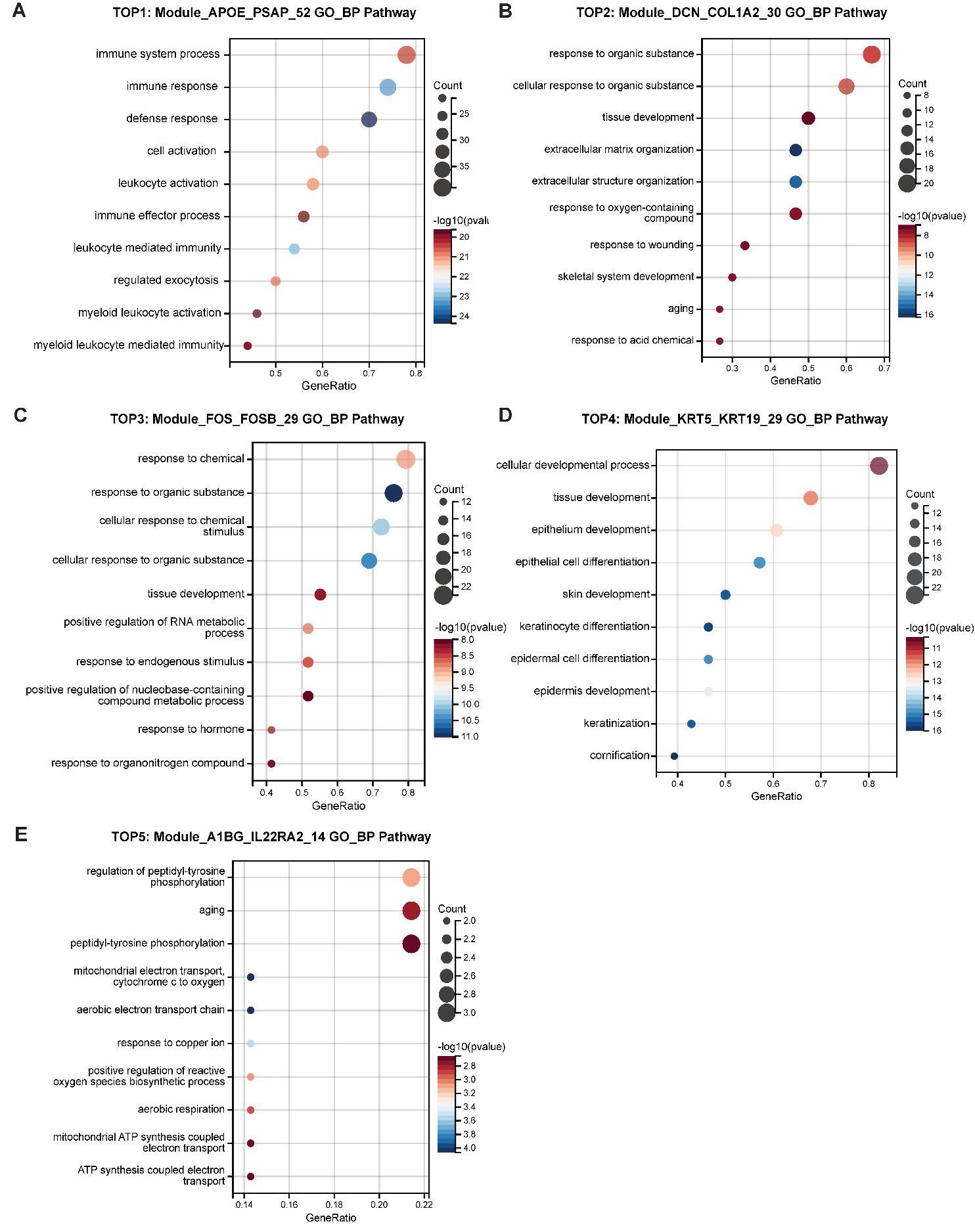


Supplementary Figure. S5 GO analysis enriched the functional pathways of the top 5 gene modules. (A) Functional pathways of the APOE_PSAP_52. (B) Functional pathways of the DCN_COL1A2_30. (C) Functional pathways of the FOS_FOSB_29. (D) Functional pathways of the KRT5_KRT19_29. (E) Functional pathways of the A1BG_IL22RA2_14.


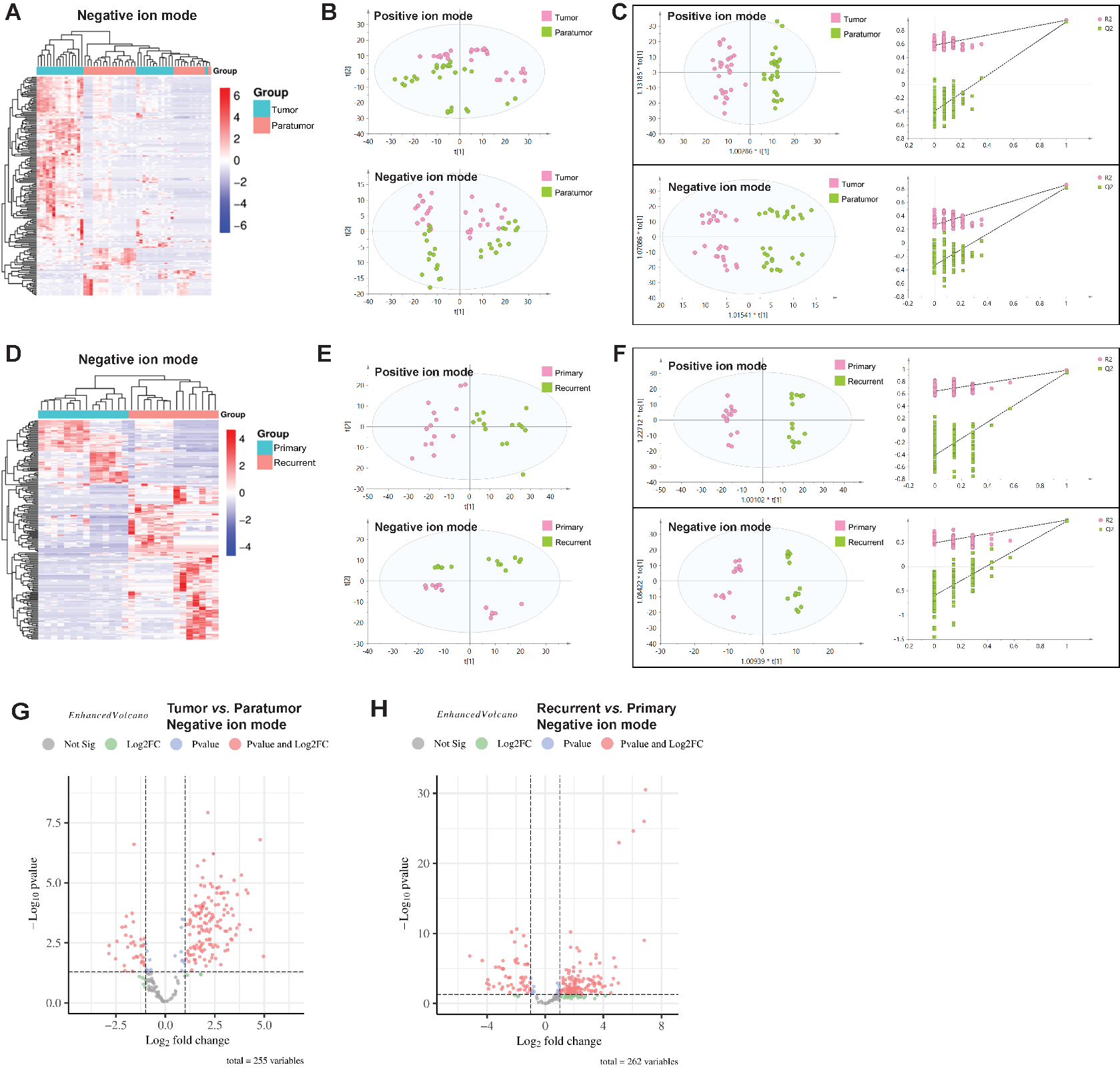
Supplementary Figure. S6 Spatially resolved metabolomics analysis in ACP by AFADESI-MSI. (A) Cluster heatmap of metabolites abundance between the tumor and paratumor tissues under the negative ion mode. (B) PCA analysis of tumor and paratumor tissue samples. (C) OPLS-DA analysis and permutation test of tumor and paratumor tissue samples. Positive ion mode: R2=0.954, Q2=0.931; Negative ion mode: R2=0.823, Q2=0.913. (D) Cluster heatmap of metabolites abundance of tumor epithelium between the primary and recurrent samples under the negative ion mode. (E) PCA analysis of primary and recurrent tumor epithelium tissue samples. (F) OPLS-DA analysis and permutation test of primary and recurrent tumor epithelium tissue samples. Positive ion mode: R2=0.985, Q2=0.951; Negative ion mode: R2=0.957, Q2=0.936. (G) Volcano plots show the total of 255 differentially enriched metabolites (VIP>1 in OPLS-DA analysis) under the negative ion mode between the tumor and paratumor tissues. 138 and 34 metabolites were significantly upregulated and downregulated (p<0.05 in the Mann–Whitney U test, |log2FC|>1) in tumor tissues, respectively. (H) Volcano plots show the total of 262 differentially enriched metabolites (VIP>1 in OPLS-DA analysis) under the negative ion mode between the primary and recurrent tumor epithelium tissues. 132 and 52 metabolites were significantly upregulated and downregulated (p<0.05 in the Mann–Whitney U test, |log2FC|>1) in recurrent tumor epithelium tissues, respectively.


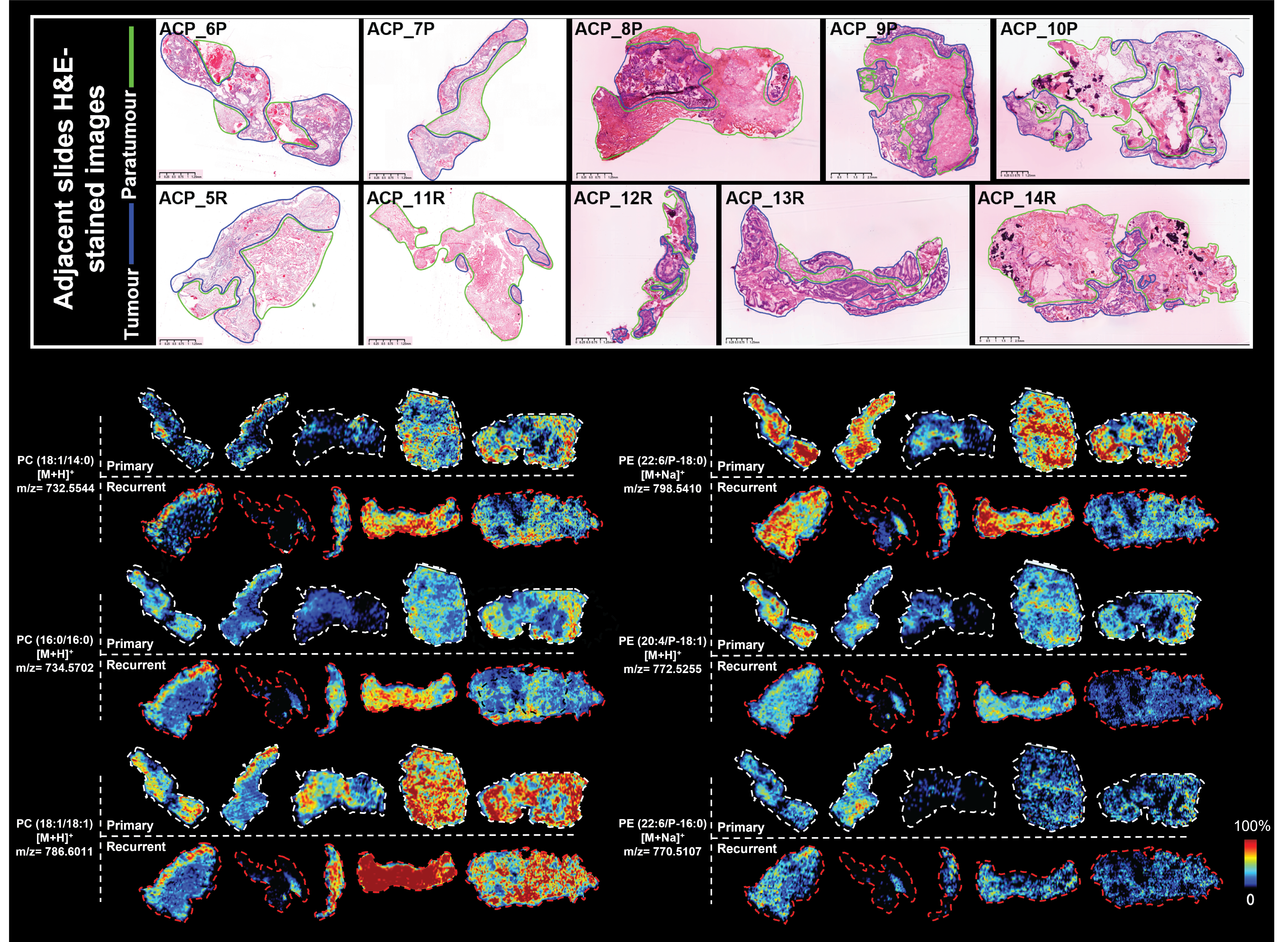
Supplementary Figure. S7 The ion intensities of saturated/unsaturated PCs and PEs, were significantly increased in tumor tissues than in paratumor tissues and in recurrent tumor epithelium tissues than in primary tumor epithelium tissues (intensity in color scale is relative value).


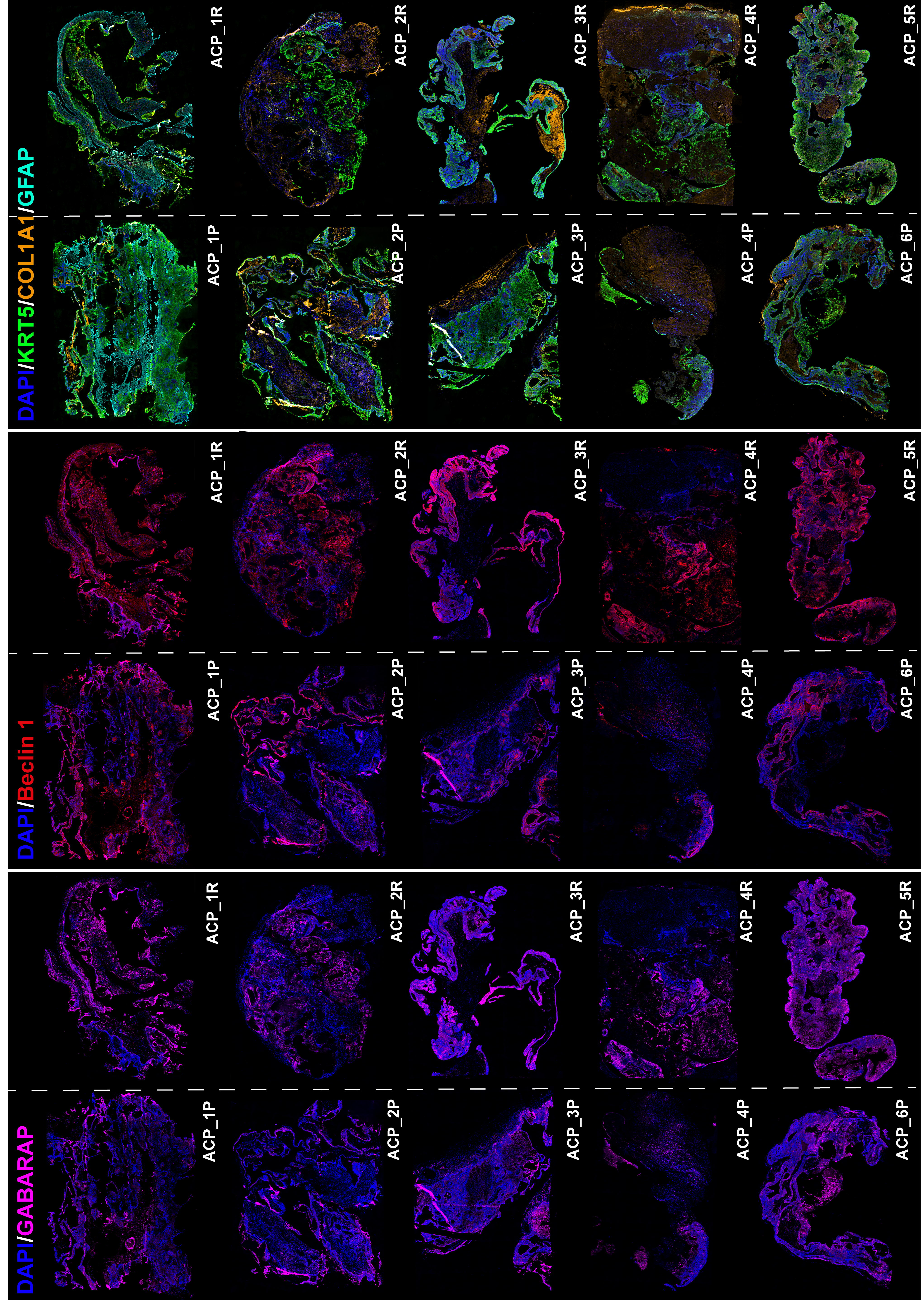
Supplementary Figure. S8 Verification of the expressions of KRT5, COL1A1, GFAP, Beclin1, and GABARAP in 10 ACP tissues by multi-IHC staining.

**Supplementary** **Table S1. Patient and sample information of single-cell spatial transcriptomics, spatially resolved metabolomics, and multiplex immunohistochemical analysis.**

| **Patient** | **Gender** | **Age** | **Diagnosis** | **Presentation** | **Sample ID** | **Sample type** | **Test item** |
| --- | --- | --- | --- | --- | --- | --- | --- |
| ACP_1 | Male | 6 | ACP | Primary | ACP_1P | Tumor tissue | SCST, multi-IHC |
| ACP_1 | Male | 8 | ACP | Recurrent | ACP_1R | Tumor tissue | SCST, multi-IHC |
| ACP_2 | Male | 53 | ACP | Primary | ACP_2P | Tumor tissue | SCST, multi-IHC |
| ACP_2 | Male | 57 | ACP | Recurrent | ACP_2R | Tumor tissue | SCST, multi-IHC |
| ACP_3 | Male | 6 | ACP | Primary | ACP_3P | Tumor tissue | SCST, multi-IHC |
| ACP_3 | Male | 12 | ACP | Recurrent | ACP_3R | Tumor tissue | SCST, multi-IHC |
| ACP_4 | Female | 2 | ACP | Primary | ACP_4P | Tumor tissue | SCST, multi-IHC |
| ACP_4 | Female | 5 | ACP | Recurrent | ACP_4R | Tumor tissue | SCST, multi-IHC |
| ACP_5 | Female | 56 | ACP | Recurrent | ACP_5R | Tumor tissue | SCST, SM,  multi-IHC |
| ACP_6 | Male | 56 | ACP | Primary | ACP_6P | Tumor tissue | SCST, SM,  multi-IHC |
| ACP_7 | Male | 1 | ACP | Primary | ACP_7P | Tumor tissue | SM |
| ACP_8 | Male | 6 | ACP | Primary | ACP_8P | Tumor tissue | SM |
| ACP_9 | Male | 3 | ACP | Primary | ACP_9P | Tumor tissue | SM |
| ACP_10 | Male | 5 | ACP | Primary | ACP_10P | Tumor tissue | SM |
| ACP_11 | Male | 12 | ACP | Recurrent | ACP_11R | Tumor tissue | SM |
| ACP_12 | Male | 9 | ACP | Recurrent | ACP_12R | Tumor tissue | SM |
| ACP_13 | Male | 5 | ACP | Recurrent | ACP_13R | Tumor tissue | SM |
| ACP_14 | Male | 7 | ACP | Recurrent | ACP_14R | Tumor tissue | SM |

SCST: single-cell spatial transcriptomics; SM: spatially resolved metabolomics; multi-IHC: multiplex immunohistochemical

**Supplementary Table S2. Patient and sample information of untargeted and targeted metabolomics analysis.**

| **Patient** | **Gender** | **Age** | **Diagnosis** | **Presentation** | **Sample ID** | **Sample type** | **Test item** |
| --- | --- | --- | --- | --- | --- | --- | --- |
| ACP_8 | Male | 6 | ACP | Primary | Cystfluid_1 | Cystic fluid | UM |
| ACP_8 | Male | 6 | ACP | Primary | Plasma_1 | Plasma | UM |
| ACP_9 | Male | 3 | ACP | Primary | Cystfluid_2 | Cystic fluid | UM |
| ACP_9 | Male | 3 | ACP | Primary | Plasma_2 | Plasma | UM |
| ACP_15 | Male | 28 | ACP | Primary | Cystfluid_3 | Cystic fluid | UM |
| ACP_15 | Male | 28 | ACP | Primary | Plasma_3 | Plasma | UM |
| ACP_16 | Male | 11 | ACP | Primary | Cystfluid_4 | Cystic fluid | UM |
| ACP_16 | Male | 11 | ACP | Primary | Plasma_4 | Plasma | UM |
| ACP_17 | Famale | 11 | ACP | Primary | Cystfluid_5 | Cystic fluid | UM |
| ACP_17 | Famale | 11 | ACP | Primary | Plasma_5 | Plasma | UM |
| ACP_18 | Famale | 19 | ACP | Primary | Cystfluid_6 | Cystic fluid | UM |
| ACP_5 | Female | 56 | ACP | Recurrent | Cystfluid_7 | Cystic fluid | UM |
| ACP_19 | Male | 53 | ACP | Recurrent | Cystfluid_8 | Cystic fluid | UM |
| ACP_19 | Male | 53 | ACP | Recurrent | Plasma_6 | Plasma | UM |
| ACP_20 | Male | 13 | ACP | Recurrent | Cystfluid_9 | Cystic fluid | UM |
| ACP_20 | Male | 13 | ACP | Recurrent | Plasma_7 | Plasma | UM |
| ACP_21 | Famale | 10 | ACP | Recurrent | Cystfluid_10 | Cystic fluid | UM |
| ACP_21 | Famale | 10 | ACP | Recurrent | Plasma_8 | Plasma | UM |
| ACP_22 | Male | 40 | ACP | Recurrent | Cystfluid_11 | Cystic fluid | UM |
| ACP_22 | Male | 40 | ACP | Recurrent | Plasma_9 | Plasma | UM |
| ACP_8 | Male | 6 | ACP | Primary | Cystfluid_1 | Cystic fluid | TM |
| ACP_8 | Male | 6 | ACP | Primary | Plasma_1 | Plasma | TM |
| ACP_9 | Male | 3 | ACP | Primary | Cystfluid_2 | Cystic fluid | TM |
| ACP_9 | Male | 3 | ACP | Primary | Plasma_2 | Plasma | TM |
| ACP_10 | Male | 5 | ACP | Primary | Cystfluid_12 | Cystic fluid | TM |
| ACP_10 | Male | 5 | ACP | Primary | Plasma_10 | Plasma | TM |
| ACP_13 | Male | 5 | ACP | Recurrent | Cystfluid_13 | Cystic fluid | TM |
| ACP_13 | Male | 5 | ACP | Recurrent | Plasma_11 | Plasma | TM |
| ACP_23 | Famale | 20 | ACP | Recurrent | Cystfluid_14 | Cystic fluid | TM |
| ACP_23 | Famale | 20 | ACP | Recurrent | Plasma_12 | Plasma | TM |
| ACP_24 | Famale | 19 | ACP | Recurrent | Cystfluid_15 | Cystic fluid | TM |
| ACP_24 | Famale | 19 | ACP | Recurrent | Plasma_13 | Plasma | TM |

UM: untargeted metabolomics; TM: targeted metabolomics

**Supplementary Table S3. Antibody and fluorescence marker information of multi-IHC**

| **Primary antibody/ Secondary antibody** | **Manufacturer** | **Product code** | **Species** | **Dilution ratio** | **Fluorescence marker** |
| --- | --- | --- | --- | --- | --- |
| KRT5 | CST | 71536 | Rabbit | 1:500 | 488 |
| COL1A1 | CST | 72026 | Rabbit | 1:100 | 594 |
| GFAP | CST | 3670 | Mouse | 1:500 | 450 |
| Beclin1 | Abcam | ab210498 | Rabbit | 1:100 | CY3 |
| GABARAP | Abcam | ab10936 | Rabbit | 1:100 | CY5 |
| HRP-labeled goat anti-rabbit antibody | SeraCare | 5220-0336 | / | 1:400 | / |
| HRP-labeled goat anti-mouse antibody | SeraCare | 5220-0341 | / | 1:400 | / |

**Supplementary Table S4. Running overview of CosMx SMI single-cell spatial transcriptomics sequencing.**

| **Parameters** | **Detection results** |
| --- | --- |
| Gene Panel Used | Human RNA 6k Discovery |
| Number of Cells | 296304 |
| Number of FOV | 386 |
| Total Transcripts Detected | 180714696 |
| Mean Transcripts per cell | 609.9 |
| Mean Features per cell | 438.85 |
| Mean Transcripts per FOV | 468172.79 |
| Total Negative Probs Detected | 280878 |
| Mean Negative Probs per cell | 0.95 |
| Total Falsecode Detected | 331209 |
| Mean Falsecode per cell | 1.12 |
| % Counts on Targeted Genes | 99.66 |
| % Cells Passed QC | 79.1 |
